# Identification of the New In Vivo Metabolites of Ilaprazole in Rat Plasma after Oral Administration by LC-MS: In Silico Prediction of the H^+^/K^+^-ATPase Inhibitor

**DOI:** 10.3390/molecules26020459

**Published:** 2021-01-16

**Authors:** Guiqiu Zhang, Kaijing Guo, Pengfei Wang, Yingbo Shan, Chen Ma

**Affiliations:** Institute of Materia Medica, Chinese Academy of Medical Sciences and Peking Union Medical College, Beijing 100050, China; zhangguiqiu@imm.ac.cn (G.Z.); guokaijing@imm.ac.cn (K.G.); pengfeiwang@imm.ac.cn (P.W.); shanyingbo@imm.ac.cn (Y.S.)

**Keywords:** ilaprazole, proton pump inhibitors, metabolite identification, bioactivity prediction, LC-MS

## Abstract

Ilaprazole is a proton pump inhibitor used to treat digestive diseases. In this study, blood samples were collected after oral administration of ilaprazole and prepared by liquid–liquid extraction. The metabolites of ilaprazole were detected by liquid chromatography–high-resolution mass spectrometry (LC-HRMS) and LC-MS^n^. A total of twelve in vivo metabolites were detected in rat plasma and six new metabolites of ilaprazole, including one reductive metabolite with sulfide (**M3**), two hydroxylated metabolites with sulfoxide (**M7** and **M8**), and three oxidative metabolites with sulfone (**M9**, **M11**, and **M12**), were identified. The possible metabolic pathways of ilaprazole and the fragmentation behaviors of its metabolites were elucidated. The result of the in silico prediction indicates that all the new metabolites showed the potential ability to inhibit H^+^/K^+^-ATPase activity.

## 1. Introduction

Ilaprazole is a proton pump inhibitor (PPI) that was approved for the treatment of digestive diseases such as gastroesophageal reflux disease, peptic ulcers, and helicobacter pylori infections [1,2,3,4,5,6]. Ilaprazole selectively accumulates in the gastric parietal cells and then activates into sulfites and sulfonamides, which can irreversibly inhibit H^+^/K^+^-ATPase activity to suppress gastric acid secretion [5]. Compared with traditional PPIs, ilaprazole provides a better suppression of acid, especially during the night, which can satisfy the clinical requirements of relieving symptoms for a long time [7].

**Figure 1:**
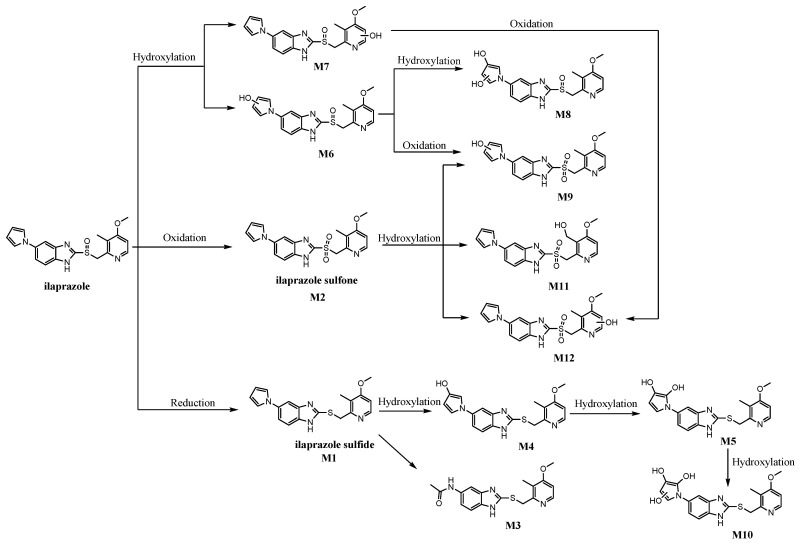
The metabolites of ilaprazole and the possible metabolic pathways in rats.

Drug metabolism is an important in vivo biotransformation process, displaying a significant role in the study of drug efficacy and safety [8]. The metabolism of ilaprazole is mainly mediated by CYP450 [9,10]. The metabolites of ilaprazole, **M1** and **M2**, have been detected in human and rat plasma by liquid chromatography coupled with mass spectrometry (LC-MS) [11,12]. Four metabolites of ilaprazole (including **M1**, **M4**, and **M5**) have been detected in human urinary samples [13]. Nine metabolites of ilaprazole (including **M1**, **M2**, **M4**, **M5**, **M6**, and **M10**) have been identified in human liver microsome (HLM) incubation samples with ilaprazole [14]. As far as we know, there are few studies on the bioactivity of the metabolites of ilaprazole. It is thus significant to identify more ilaprazole metabolites and predict their bioactivities. LC-MS analytical technology is widely used in the identification of metabolites with high sensitivity [15,16]. The present study aims to identify in vivo metabolites of ilaprazole in rat plasma by LC-MS. Blood samples were collected after oral administration of ilaprazole. After being prepared by liquid–liquid extraction, the samples were detected via liquid chromatography–tandem high-resolution mass spectrometry (LC-HRMS/MS).The fragmentation behaviors of ilaprazole and its metabolites have been elucidated by LC-MS^n^ and LC-HRMS/MS. Twelve metabolites were identified in rat plasma and the possible metabolic pathways of ilaprazole in a rat model are described in Figure 1. The potential bioactivity of the new metabolites of ilaprazole was predicted by the Prediction of Activity Spectra for Substances (PASS) [17] software product.

## 2. Results and Discussion

### 2.1. Detection of Ilaprazole and Its Metabolites

The possible metabolites of ilaprazole were determined by the following strategies. The MS/MS spectrum of ilaprazole was obtained by enhanced product ion (EPI) scanning to obtain the characteristic product ions of ilaprazole. The software LightSight was utilized to build a predictive method, the multiple reaction monitoring (MRM)–information-dependent acquisition (IDA)–EPI method, according to the product ions of ilaprazole. The characteristic fragment ion at *m/z* 184 was used as a precursor ion (Prec) -IDA-EPI mode for the Analyst 1.6 software. Some oxidative metabolites with a sulfone structure show the loss of SO_2_ (64 Da) in the fragmentation pathway [18], so the neutral loss (NL)-IDA-EPI mode of Analyst 1.6 software was selected to monitor the oxidative metabolites, including the sulfone in the structure. The HRMS/MS spectrum and the accurate mass data were obtained by LC-HRMS/MS.

**Figure 2:**
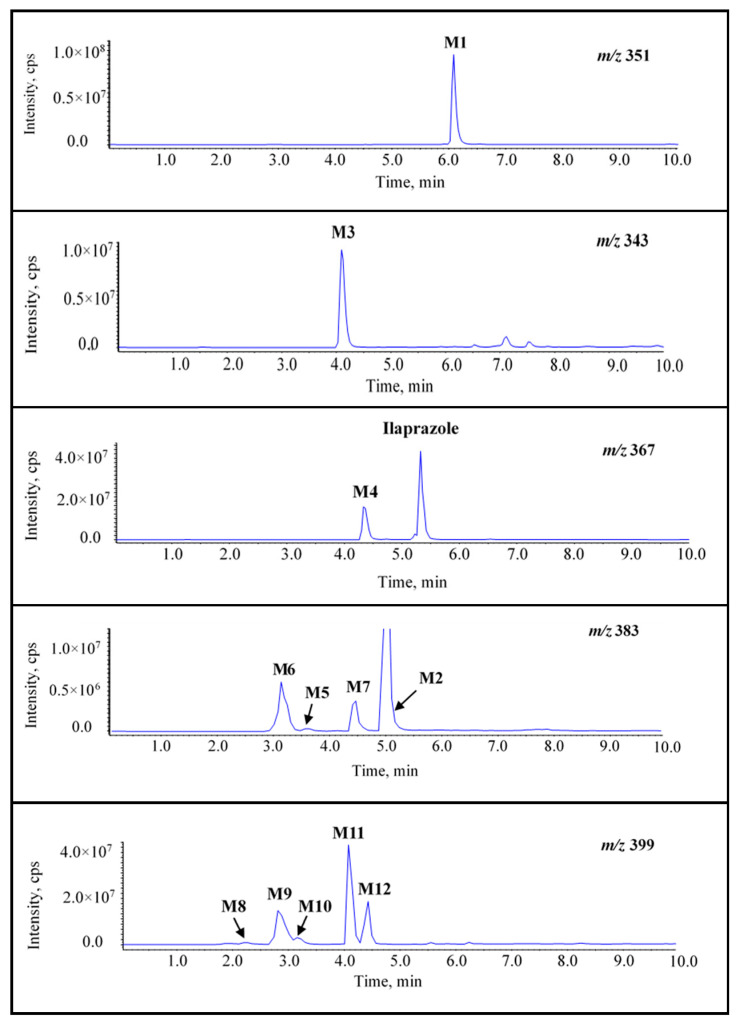
The enhanced product ion (EPI) chromatograms of ilaprazole and twelve metabolites.

Twelve metabolites were detected in the rats’ plasma samples and named **M1**–**M12**. **M3**, **M7**, **M8**, **M9**, **M11**, and **M12** are the new metabolites of ilaprazole. Their structures were characterized via high-resolution mass data. The EPI chromatograms of ilaprazole and the twelve metabolites are described in Figure 2. The accurate masses of the protonated molecule and fragment ions are shown in Supplementary Material Table S1.

### 2.2. Characteristics of Ilaprazole, **M1**, and **M2**

The metabolites of ilaprazole can be divided into three structural types, including reductive metabolites (ilaprazole sulfide derivatives), oxidative metabolites (ilaprazole sulfone derivatives), and hydroxylated metabolites (sulfoxide derivatives). The analysis of the mass spectra of ilaprazole, **M1**, and **M2** was essential for identifying the metabolites of ilaprazole.

#### 2.2.1. Ilaprazole

**Figure 3:**
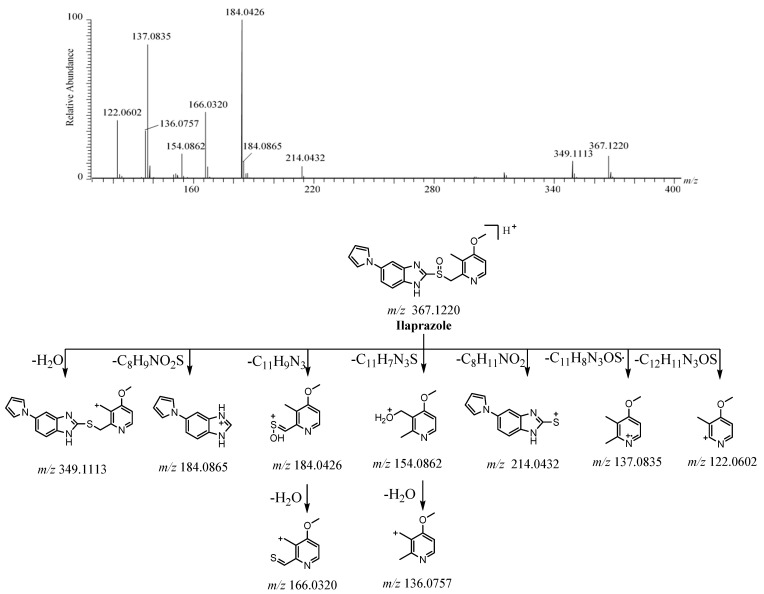
The tandem high-resolution mass spectrometry (HRMS/MS) spectrum and the possible fragmentation pathway of ilaprazole.

The [M + H]^+^ of ilaprazole was observed at *m*/*z* 367.1220 [C_19_H_19_N_4_O_2_S]^+^ (Supplementary Material Table S1). The dominant fragment ions at *m/z* 184.0865 (C_11_H_10_N_3_^+^) and *m*/*z* 184.0426 (C_8_H_10_NO_2_S^+^) were formed from the fragmentation between the sulfur atom and benzimidazole [13]. Then, the ion at *m*/*z* 184.0426 was followed by the elimination of H_2_O to form the ion at *m*/*z* 166. The disconnection of the bond between the C-S bond of [M + H]^+^ produced the fragment ions at *m*/*z* 214 and *m*/*z* 154 via rearrangement. The ion at *m*/*z* 154 was followed by the elimination of H_2_O to form the ion at *m*/*z* 136. The fragment ions at *m*/*z* 349, *m*/*z* 137, and *m*/*z* 122 were created due to the loss of H_2_O, C_11_H_8_N_3_OS·, and C_12_H_11_N_3_OS from [M + H]^+^, respectively. The HRMS/MS spectrum is shown in Figure 3. The [M + H]^+^, fragment ions, and the retention time of ilaprazole were the same as in the reference standards. These proposed fragment ions can be supported by MS^n^ (Supplementary Material Figure S1A). The possible fragmentation pathway of ilaprazole is depicted in Figure 3.

#### 2.2.2. **M1**

**Figure 4:**
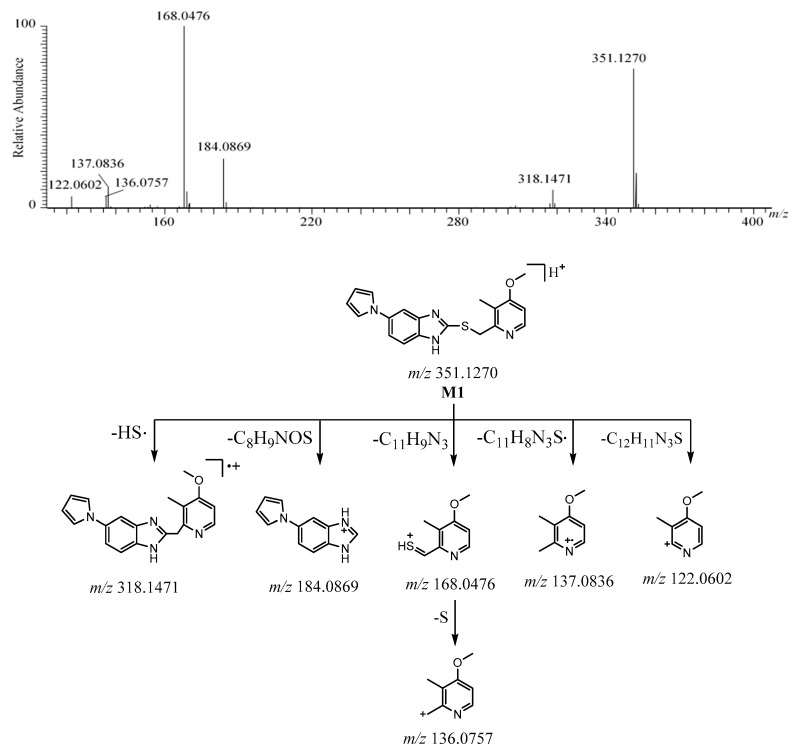
The HRMS/MS spectrum and the possible fragmentation pathway of M1.

The [M + H]^+^ of **M1** was observed at *m*/*z* 351.1270 [C_19_H_19_N_4_OS]^+^ (Supplementary Material Table S1). The protonated molecule at *m*/*z* 351 was 16 Da less than ilaprazole. The characteristic fragment ion at *m*/*z* 318 was produced to lose 33 Da (HS) from [M + H]^+^, which is a typical fragmentation for pyrrole-substituted benzimidazoles with a –CH_2_–S– link to the pyridine ring [19]. It could be deduced that the sulfoxide of ilaprazole was reduced to sulfide. The fragmentation between the sulfur atom and benzimidazole of [M + H]^+^ formed ions at *m*/*z* 184.0869 and *m*/*z* 168.0476. The ion at *m*/*z* 168 was 16 Da less than *m*/*z* 184.0426 in ilaprazole. The ion at *m*/*z* 168 was followed by formation of a fragment ion at *m*/*z* 136 after losing S. The ions at *m*/*z* 137 and *m*/*z* 122 arose from [M + H]^+^ after losing C_11_H_8_N_3_S· and C_12_H_11_N_3_S, respectively. The ions at *m*/*z* 137, *m*/*z* 136, and *m*/*z* 122 were also observed for ilaprazole, which showed that there was the same skeletal structure in the substituted pyridine moiety. The ion at *m*/*z* 184.0869 was the same as ilaprazole, which suggests that there was the same skeletal structure in the substituted benzimidazole moiety. The [M + H]^+^, fragment ions, and the retention time of **M1** were the same as the reference standards of ilaprazole sulfide. These proposed fragment ions can be supported by MS^n^ (Supplementary Material Figure S1B). Based on the above, the possible structure of **M1** was deduced as ilaprazole sulfide. The HRMS/MS spectrum and the possible fragmentation pathway of **M1** are depicted in Figure 4.

#### 2.2.3. **M2**

**Figure 5:**
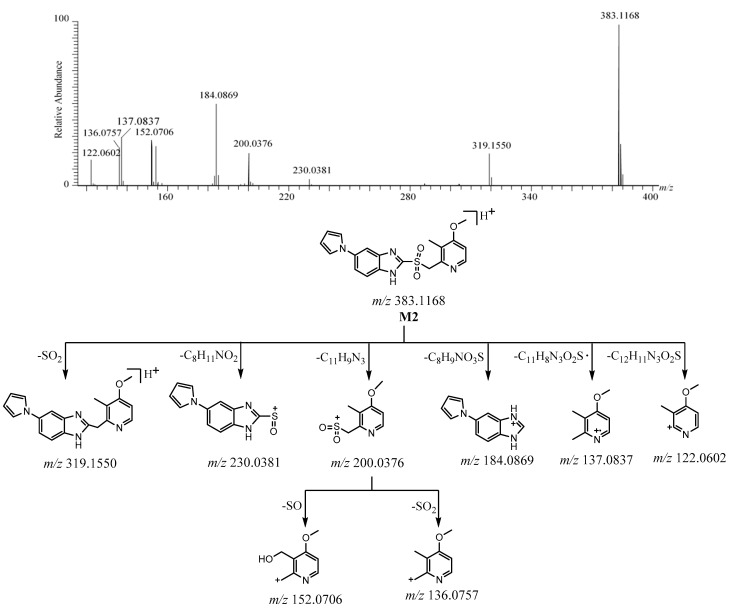
The HRMS/MS spectrum and the possible fragmentation pathway of M2.

The [M + H]^+^ of **M2** was detected at *m*/*z* 383.1168 [C_19_H_19_N_4_O_3_S]^+^ (Supplementary Material Table S1). The protonated molecule at *m*/*z* 383 was 16 Da more than ilaprazole. The characteristic fragment ion of **M2** at *m*/*z* 319 was produced to lose 64 Da (SO_2_) from [M + H]^+^, as suggested by the existence of sulfone [18]. The disconnection between benzimidazole and the sulfur atom of [M + H]^+^ led to the ions at *m*/*z* 200.0376 (16 Da more than *m*/*z* 184.0426 in ilaprazole) and *m*/*z* 184.0869 (same as ilaprazole). The ion at *m*/*z* 200 was followed by the elimination of SO and SO_2_ to yield the ions at *m*/*z* 152 and *m*/*z* 136, respectively. The ions at *m*/*z* 230, *m*/*z* 137, and *m*/*z* 122 arose from [M + H]^+^ after losing C_8_H_11_NO_2_, C_11_H_8_N_3_O_2_S·, and C_12_H_11_N_3_O_2_S, respectively. The ions at *m*/*z* 137, *m*/*z* 136, and *m*/*z* 122 were also observed for ilaprazole, which suggested that there were no changes in the substituted pyridine ring. The ion at *m*/*z* 184.0869 was similar to ilaprazole, which suggests that there was the same skeletal structure in the substituted benzimidazole moiety. The HRMS/MS spectrum is shown in Figure 5. The [M + H]^+^, fragment ions, and the retention time of **M2** were the same as the reference standards of ilaprazole sulfone. These proposed fragment ions can be supported by MS^n^ (Supplementary Material Figure S1C). Based on the above, the possible structure of **M2** was deduced as ilaprazole sulfone. The possible fragmentation pathway of **M2** is also depicted in Figure 5.

### 2.3. Identification of the New Metabolites of Ilaprazole

#### 2.3.1. **M3**

**Figure 6:**
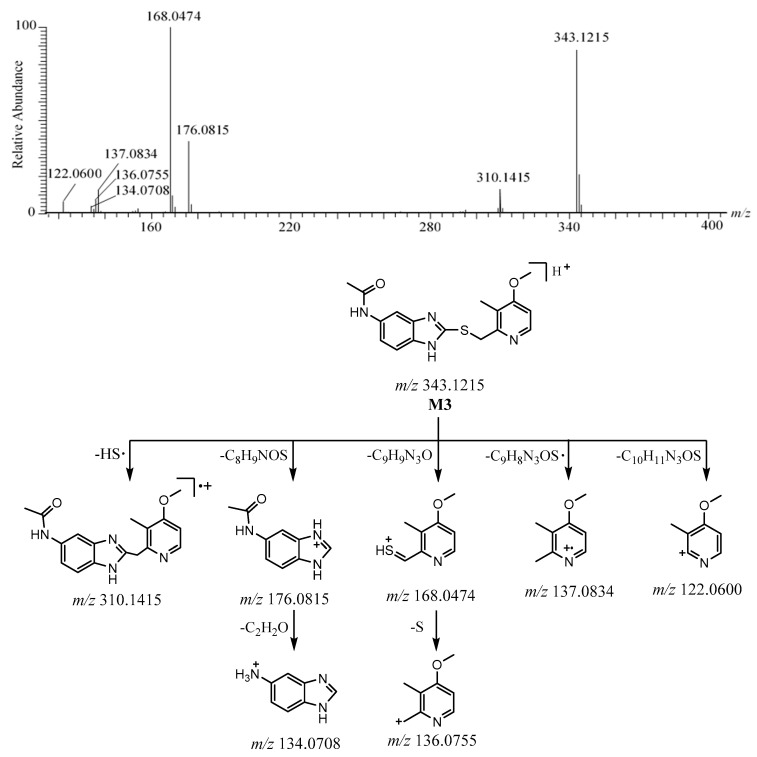
The HRMS/MS spectrum and the possible fragmentation pathway of M3.

The [M + H]^+^ of **M3** was observed at *m*/*z* 343.1215 [C_17_H_19_N_4_O_2_S]^+^ (Supplementary Material Table S1). The protonated molecule at *m*/*z* 343 was 24 Da less than ilaprazole. The characteristic fragment ion at *m*/*z* 310 was formed to lose 33 Da (HS) from [M + H]^+^, which indicated the existence of a sulfide bond, as with **M1**. The dominant fragment ions at *m*/*z* 176.0815 (C_9_H_10_N_3_O^+^) and *m*/*z* 168.0474 (C_8_H_10_NOS^+^) formed as a result of the fragmentation between the sulfur atom and benzimidazole of [M + H]^+^. The fragment ion at *m*/*z* 176 was followed by the production of the ion at *m*/*z* 134 by the elimination of 42 Da (C_2_H_2_O). It could be deduced that there may be an amide bond in **M3**. The fragment ion at *m*/*z* 168 was followed by losing S to form a fragment ion at *m*/*z* 136. The ions at *m/z* 137 and *m*/*z* 122 arose from [M + H]^+^ after losing C_9_H_8_N_3_OS· and C_10_H_11_N_3_OS, respectively. The fragment ions at *m*/*z* 168, *m*/*z* 137, *m*/*z* 136, and *m*/*z* 122 were also observed for **M1**, as there were no changes in the substituted pyridine ring. The HRMS/MS spectrum is described in Figure 6. These proposed fragment ions can be supported by MS^n^ (Supplementary Material Figure S1D). Based on the above, the possible structure and the possible fragmentation pathway of **M3** are depicted in Figure 6.

#### 2.3.2. **M7**

**Figure 7:**
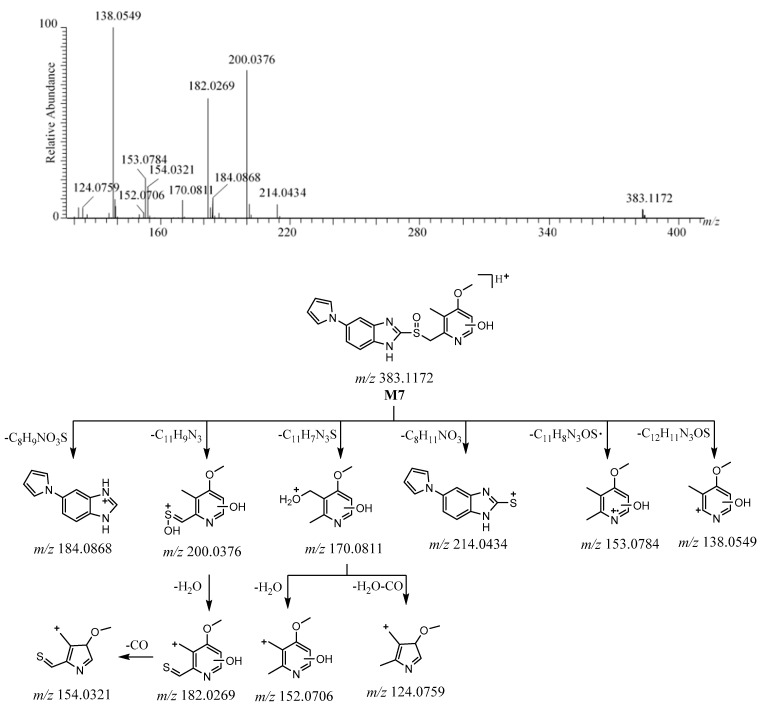
The HRMS/MS spectrum and the possible fragmentation pathway of **M7**.

The [M + H]^+^ of **M7** was observed at *m*/*z* 383.1172 [C_19_H_19_N_4_O_3_S]^+^ (Supplementary Material Table S1). The protonated molecule of **M7** was 16 Da more than ilaprazole. The ions at *m*/*z* 200.0376 (C_8_H_10_NO_3_S^+^) and *m*/*z* 184.0868 (C_11_H_10_N_3_^+^) are believed to be the products of the fragmentation between the sulfur atom and benzimidazole; the ion at *m*/*z* 200 was followed by the elimination of H_2_O to form the ion at *m*/*z* 182, which lost CO to yield the ion at *m*/*z* 154. The disconnection of the bond between the C-S bond of [M + H]^+^ produced the fragment ions at *m*/*z* 214 and *m*/*z* 170 via rearrangement, and the ion at *m*/*z* 170 was followed by the loss of H_2_O and CO+H_2_O to form the ions at *m*/*z* 152 and *m*/*z* 124, respectively. This suggests that a hydroxyl group was added in the pyrrole-substituted benzimidazole moiety. The fragment ions at *m*/*z* 153 and *m*/*z* 138 arose from [M + H]^+^ after losing C_11_H_8_N_3_OS· and C_12_H_11_N_3_OS, respectively. The produced ions at *m*/*z* 214 and *m*/*z* 184.0869 were the same as ilaprazole, which suggests that there are no changes in the substituted benzimidazole moiety. The fragment ions at *m*/*z* 200, *m*/*z* 182, *m*/*z* 170, *m*/*z* 152, *m*/*z* 153, and *m*/*z* 138 were 16 Da more than those ions at *m*/*z* 184.0426, *m*/*z* 166, *m*/*z* 154, *m*/*z* 136, *m*/*z* 137, and *m*/*z* 122 of ilaprazole, respectively, which indicates that a hydroxyl group was added in the pyridine moiety, and the position of the hydroxyl group could not be determined. These proposed fragment ions can be supported by MS^n^ (Supplementary Material Figure S1H). The HRMS/MS spectrum and the possible fragmentation pathway of **M7** are depicted in Figure 7.

#### 2.3.3. **M8**

**Figure 8:**
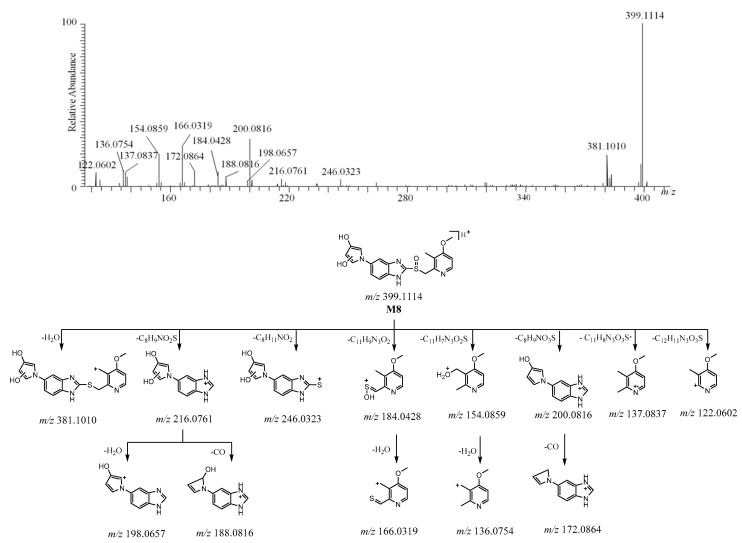
The HRMS/MS spectrum and the possible fragmentation pathway of M8.

The [M + H]^+^ of **M8** was detected at *m*/*z* 399.1114 [C_19_H_19_N_4_O_4_S]^+^ (Supplementary Material Table S1). The protonated molecule of **M8** was 32 Da more than ilaprazole. The ions at *m*/*z* 216.0761 (C_11_H_10_N_3_O_2_^+^) and *m*/*z* 184.0428 (C_8_H_10_NO_2_S^+^) are believed to be the products of the fragmentation between the sulfur atom and benzimidazole, and the ion at *m*/*z* 184.0428 was followed by the elimination of H_2_O to form the ion at *m*/*z* 166. The disconnection of the bond between the C-S bond of [M + H]^+^ produced the fragment ions at *m*/*z* 246 and *m*/*z* 154 via rearrangement, and the ion at *m*/*z* 154 was followed by the elimination of H_2_O to form the ion at *m*/*z* 136. The fragment ions at *m*/*z* 381, *m*/*z* 200, *m*/*z* 137, and *m*/*z* 122 arose from [M + H]^+^ after losing H_2_O, C_8_H_9_NO_3_S, C_11_H_8_N_3_O_3_S·, and C_12_H_11_N_3_O_3_S, respectively. After losing CO from *m*/*z* 200, the ion at *m*/*z* 172 was created. The fragment ions at *m*/*z* 246 and *m*/*z* 216 were 32 Da more than those ions at *m*/*z* 214 and *m*/*z* 184.0865 of ilaprazole, respectively. The ion at *m*/*z* 216 lost H_2_O and CO to yield the ions at *m*/*z* 198 and *m*/*z* 188 respectively, which suggests that two hydroxyl groups were added in the pyrrole-substituted benzimidazole moiety, and the positions of the hydroxyl groups could not be determined. The fragment ions at *m*/*z* 184.0428, *m*/*z* 137, *m*/*z* 136, and *m*/*z* 122 were also observed for ilaprazole, as there are no changes in the substituted pyridine moiety. These proposed fragment ions can be supported by MS^n^ (Supplementary Material Figure S1I). The HRMS/MS spectrum and the possible fragmentation pathway of M8 are described in Figure 8.

#### 2.3.4. **M9**

**Figure 9:**
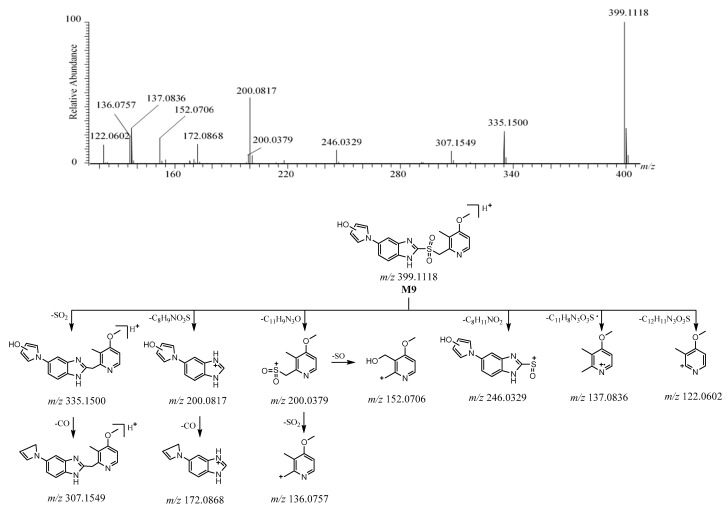
The HRMS/MS spectrum and the possible fragmentation pathway of M9.

The [M + H]^+^ of **M9** was detected at *m*/*z* 399.1118 [C_19_H_19_N_4_O_4_S]^+^ (Supplementary Material Table S1). The protonated molecule of **M9** was 32 Da more than ilaprazole. The characteristic fragment ion of **M9** at *m*/*z* 335 was produced to lose 64 Da (SO_2_) from [M + H]^+^, which suggests the existence of a sulfone, as with **M2**. Then, the ion at *m*/*z* 335 lost CO to yield the ion at *m*/*z* 307. The ions at *m*/*z* 200.0817 and *m*/*z* 200.0379 are believed to be the product ions from the fragmentation between the sulfur atom and benzimidazole of [M + H]^+^, and the ion at *m*/*z* 200.0379 was followed by the elimination of SO and SO_2_ to form the ions at *m*/*z* 152 and *m*/*z* 136, respectively. The ion at *m*/*z* 172 arose from the fragment ion at *m*/*z* 200.0817 after losing CO. The fragment ions at *m*/*z* 246, *m*/*z* 137, and *m*/*z* 122 arose from [M + H]^+^ after losing C_8_H_11_NO_2_, C_11_H_8_N_3_O_3_S·, and C_12_H_11_N_3_O_3_S, respectively. The product ions at *m*/*z* 246 and *m*/*z* 200.0817 were 16 Da more than those ions at *m*/*z* 230 and *m*/*z* 184.0865 of **M2**, respectively. A subsequent CO loss from *m*/*z* 335 and *m*/*z* 200 led to the ions at *m*/*z* 307 and *m*/*z* 172, respectively, which could be due to the presence of hydroxyl groups in the pyrrole-substituted benzimidazole moiety, and the positions of the hydroxyl groups could not be determined. The fragment ions at *m*/*z* 200.0379, *m*/*z* 152, *m*/*z* 137, *m*/*z* 136, and *m*/*z* 122 were also observed for **M2**, as they may share the same skeletal structure in the substituted pyridine moiety. These proposed fragment ions can be supported by MS^n^ (Supplementary Material Figure S1J). The HRMS/MS spectrum and the possible fragmentation pathway of **M9** are described in Figure 9.

#### 2.3.5. **M11**

**Figure 10:**
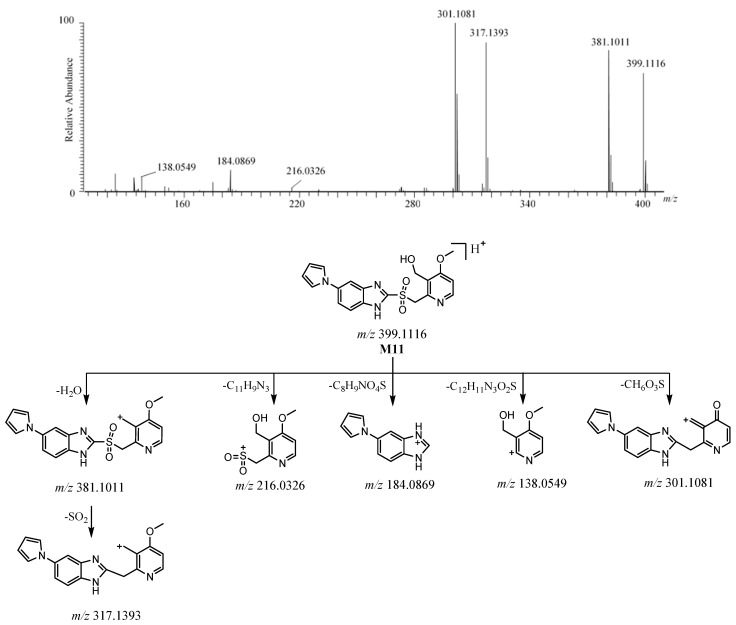
The HRMS/MS spectrum and the possible fragmentation pathway of M11.

The [M + H]^+^ of **M11** was detected at *m*/*z* 399.1116 [C_19_H_19_N_4_O_4_S]^+^ (Supplementary Material Table S1). The protonated molecule of **M11** was 32 Da more than ilaprazole. The ion at *m*/*z* at 381 formed as a result of the loss of H_2_O from [M + H]^+^, followed by the loss of SO_2_ to form the ion at *m*/*z* 317, which suggested the existence of sulfone, as with M2. The ions at *m*/*z* 184.0869 and *m*/*z* 216.0326 are believed to be the product ions of the fragmentation between the sulfur atom and benzimidazole. The ions at *m*/*z* 301 and *m*/*z* 138 came from [M + H]^+^ after the loss of CH_6_O_3_S and C_12_H_11_N_3_O_2_S, respectively. The ions at *m*/*z* 216 and *m*/*z* 138 were 16 Da more than those ions at *m*/*z* 200 and *m*/*z* 122 in **M2**, suggesting that a hydroxyl group was added in the pyridine moiety. The ion at *m*/*z* 184.0868 was also observed for **M2**, which shows that there was the same skeletal structure in the pyrrole-substituted benzimidazole moiety. These proposed fragment ions can be supported by MS^n^ (Supplementary Material Figure S1L). Based on the above, the HRMS/MS spectrum, the possible structure, and the possible fragmentation pathway of **M11** are depicted in Figure 10.

#### 2.3.6. **M12**

**Figure 11:**
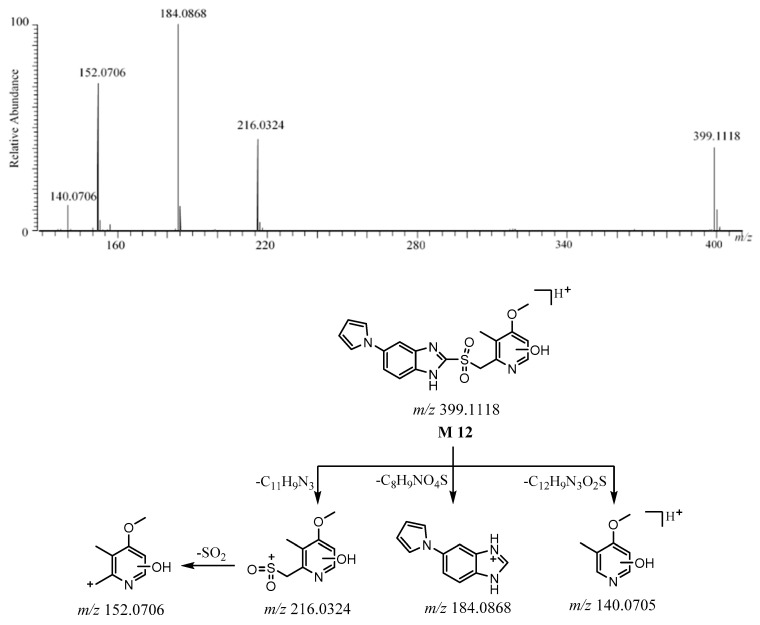
The HRMS/MS spectrum and the possible fragmentation pathway of **M12**.

The [M + H]^+^ of **M12** was detected at *m*/*z* 399.1118 [C_19_H_19_N_4_O_4_S]^+^ (Supplementary Material Table S1). The protonated molecule of **M12** was 32 Da more than ilaprazole. The ions at *m*/*z* 184.0868 and *m*/*z* 216.0324 are believed to be the product ions of the fragmentation between the sulfur atom and benzimidazole. The ion at *m*/*z* 152 came from the ion at *m*/*z* 216 after the loss of SO_2_, which suggests the existence of sulfone, as with **M2**. The ion at *m*/*z* 140 came from [M + H]^+^ after the loss of C_12_H_9_N_3_O_2_S. The fragment ion at *m*/*z* 184.0868 was also observed for **M2**, which shows that there was the same skeletal structure in the pyrrole-substituted benzimidazole moiety. The fragment ions at *m*/*z* 216 and *m*/*z* 152 were 16 Da more than those ions at *m*/*z* 200 and *m*/*z* 136 in **M2**, which shows that there was a hydroxyl group in the pyridine moiety, and the position of the hydroxyl group could not be determined. These proposed fragment ions can be supported by MS^n^ (Supplementary Material Figure S1M). The HRMS/MS spectrum and the possible fragmentation pathway of **M12** are described in Figure 11.

### 2.4. Identification of the Known Metabolites, ***M4***, ***M5***, ***M6***, and ***M10***

#### 2.4.1. **M4**

The [M + H]^+^ of M4 was observed at *m*/*z* 367.1219 [C_19_H_19_N_4_O_2_S]^+^ (Supplementary Material Table S1). The protonated molecule at *m*/*z* 367 was the same as ilaprazole. The HRMS/MS spectrum of M4 (Supplementary Material Figure S2A) showed product ions at *m*/*z* 334 (loss of HS·), *m*/*z* 200 (loss of C_8_H_9_NOS), *m*/*z* 172 (loss of C_8_H_9_NOS+CO), *m*/*z* 168 (loss of C_11_H_9_N_3_O), *m*/*z* 137 (loss of C_11_H_8_N_3_OS·), *m*/*z* 136 (loss of C_11_H_9_N_3_O+S), and *m*/*z* 122 (loss of C_12_H_11_N_3_OS). These proposed fragment ions can be supported by MS^n^ (Supplementary Material Figure S1E). The structure of **M4** (Figure 1) was consistent with the literature [13]. The possible fragmentation pathway of **M4** is described in Figure S2A.

#### 2.4.2. **M5**

The [M + H]^+^ of **M5** was observed at *m*/*z* 383.1161 [C_19_H_19_N_4_O_3_S]^+^ (Supplementary Material Table S1). The protonated molecule at *m*/*z* 383 was 16 Da more than ilaprazole. The HRMS/MS spectrum of **M5** (Supplementary Material Figure S2B) showed product ions at *m*/*z* 365 (loss of H_2_O), *m*/*z* 350 (loss of HS·), *m*/*z* 216 (loss of C_8_H_9_NOS or HS+C_8_H_8_NO), *m*/*z* 188 (loss of C_8_H_9_NOS+CO), *m*/*z* 168 (loss of C_11_H_9_N_3_O_2_), *m*/*z* 137 (loss of C_11_H_8_N_3_O_2_S), *m*/*z* 136 (loss of C_11_H_9_N_3_O_2_+S), and *m*/*z* 122 (loss of C_12_H_11_N_3_O_2_S). These proposed fragment ions can be supported by MS^n^ (Supplementary Material Figure S1F). The structure of **M5** (Figure 1) was consistent with the literature [13]. The possible fragmentation pathway of **M5** is described in Figure S2B.

#### 2.4.3. **M6**

The [M + H]^+^ of **M6** was observed at *m*/*z* 383.1169 [C_19_H_19_N_4_O_3_S]^+^ (Supplementary Material Table S1). The protonated molecule of M6 was 16 Da more than ilaprazole. The HRMS/MS spectrum of **M6** (Supplementary Material Figure S2C) showed product ions at *m*/*z* 365 (loss of H_2_O), *m*/*z* 230 (loss of C_8_H_11_NO_2_), *m*/*z* 200 (loss of C_8_H_9_NO_2_S), *m*/*z* 184 (loss of C_11_H_9_N_3_O), *m*/*z* 172 (loss of C_8_H_9_NO_2_S+CO), *m*/*z* 166 (loss of C_11_H_9_N_3_O+H_2_O), *m*/*z* 154 (loss of C_11_H_7_N_3_OS), *m*/*z* 137 (loss of C_11_H_8_N_3_O_2_S·), *m*/*z* 136 (loss of C_11_H_7_N_3_OS+H_2_O), and *m*/*z* 122 (loss of C_12_H_11_N_3_O_2_S). These proposed fragment ions can be supported by MS^n^ (Supplementary Material Figure S1G). The structure of **M6** (Figure 1) was consistent with the literature [19]. The possible fragmentation pathway of **M6** is described in Figure S2C.

#### 2.4.4. **M10**

The [M + H]^+^ of **M10** was observed at *m*/*z* 399.1115 [C_19_H_19_N_4_O_4_S]^+^ (Supplementary Material Table S1). The protonated molecule of **M10** was 32 Da more than ilaprazole. The HRMS/MS spectrum of **M10** (Supplementary Material Figure S2D) showed product ions at *m*/*z* 366 (loss of HS·), *m*/*z* 232 (loss of C_8_H_9_NOS), *m*/*z* 214 (loss of C_8_H_9_NOS+H_2_O), *m*/*z* 186 (loss of C_8_H_9_NOS+H_2_O+CO), *m*/*z* 168 (loss of C_11_H_9_N_3_O_3_), *m*/*z* 137 (loss of C_11_H_8_N_3_O_3_S·), *m*/*z* 136 (loss of C_11_H_9_N_3_O_3_+S), *m*/*z* 134 (loss of C_8_H_9_NOS+C_4_H_2_O_3_), and *m*/*z* 122 (loss of C_12_H_11_N_3_O_3_S). These proposed fragment ions can be supported by MS^n^ (Supplementary Material Figure S1K). The structure of **M10** (Figure 1) was consistent with the literature [19]. The possible fragmentation pathway of **M10** is described in Figure S2D.

### 2.5. Metabolic Pathways

CYP450 is involved in the metabolism of ilaprazole. The reaction in which sulfoxide oxidizes to sulfone was mainly mediated by CYP3A [9,10]. Twelve metabolites of ilaprazole were detected in rats after oral administration. The possible metabolic pathways are summarized as follows and are depicted in Figure 1. The ilaprazole sulfoxide reduction to sulfide formed **M1**, followed by oxidation to form **M3**, **M4**, **M5**, and **M10**. The ilaprazole sulfoxide oxidation to sulfone created **M2** and subsequently hydroxylated to form **M9**, **M11**, and **M12**. The hydroxylation reaction of ilaprazole produced **M6**, **M7**, and **M8**.

### 2.6. In Silico Bioactivity Prediction of the H^+^/K^+^-ATPase Inhibitor

The potential bioactivities of the six new in vivo metabolites of ilaprazole were predicted. All of them have shown the potential ability to inhibit H^+^/K^+^-ATPase activity (Table 1).

We have predicted the potential bioactivity of all the probable structures of **M7**, **M8, M9**, and **M12**. The probability of the bioactivity may be related to the position of the hydroxyl groups. The metabolites with a hydroxyl group in the meta-position of N in the pyridine moiety have shown a higher bioactivity probability than the metabolites with a hydroxyl group in the ortho-position of N such as **M7-1** and **M12-1**. Similarly, the metabolites with a hydroxyl group in the meta-position of N in the pyrrole ring also displayed a higher bioactivity probability, which can be confirmed by **M8-2** and **M9-1**. The bioactivity probability of **M8-2** with two meta-hydroxyl groups was more than 90%.

## 3. Materials and Methods

### 3.1. Chemicals and Reagents

Ilaprazole (>99%) was purchased from the National Institutes for Food and Drug Control (Beijing, China). Ilaprazole sulfide (≥95%) and ilaprazole sulfone (≥95%) were purchased from Shanghai Yichun Technology Co. Ltd. (Shanghai, China). Methanol (HPLC grade) and ethyl acetate (HPLC grade) were obtained from Honeywell International Inc. (Morris, NJ, USA). Ammonium acetate (HPLC grade) was obtained from Roe Scientific Inc. (St. Newark, DE, USA). Ammonia (analytical grade) was purchased from Beijing Chemical Industry Group Co. Ltd. (Beijing, China). Heparin (analytical grade) and sodium carboxymethyl cellulose (chemical grade) were purchased from Sinopharm Chemical Reagent Co. Ltd. (Shanghai, China). Deionized water was obtained from a Mill-Q Reference water purification system (Millipore, Molsheim, France).

### 3.2. Instrument and Analysis Conditions

#### 3.2.1. LC-MS^n^

For separation, the processed plasma samples were treated on a chromatography system (UFLC-20AD XR, Shimadzu, Japan). The shim-pack XR-ODS II reversed-phase column (3.0 mm × 75 mm, 2.2 μm, Shimadzu, Japan) was used in this progress. The mobile phase consisted of 10 mM ammonium acetate (pH 7, Solvent A) and methanol (Solvent B). The gradient elution was set as follows: 0–0.5 min, 50% B; 0.5–4 min, 50–85% B; 4–8 min, 85% B; 8–9 min, 85–50% B; 9–10 min, 50% B. The injection volume was 3 μL, with a flow rate of 0.4 mL/min, and the column temperature was maintained at 30 °C.

For the mass spectrometry analysis, the UFLC-20AD XR was connected to a Sciex Qtrap 5500 mass system (AB SCIEX, Framingham, MA, USA) via electrospray ionization (ESI) interface in positive ion mode and controlled by Analyst software (AB SCIEX, Framingham, MA, USA) (1.6 Version). The spectra were obtained in the EPI and MS^3^ scan mode. The typical parameters for MS scan were optimized as follows: ion spray voltage (IS), 5500 V; temperature (TEM), 550 °C; curtain gas (CUR), 30 psi; ion source gas 1 (GS1), 50 psi; ion source gas 2 (GS2), 35 psi; declustering potential (DP), 20 V; entrance potential (EP), 10 V; collision energy (CE), 25–40 V; and collision cell exit potential (CXP), 10 V. High-purity nitrogen (99.9%) was used to supply the MS.

#### 3.2.2. LC-HRMS

The separation condition was the same as in Section 3.2.1. The extracts were separated using the Dionex Ultimate 3000 (Dionex, Germering, Germany) system and analyzed using a Q Exactive orbitrap mass spectrometer (Thermo Scientific Fisher, San Jose, CA, USA). The ESI source was operated in positive mode using a parallel reaction monitoring (PRM) scan with the following parameters. Spray voltage, 3500 V; capillary temperature, 300 °C; sheath gas, 45 arb; aux gas, 10 arb; scan range, *m*/*z* 50–850; resolution, 17500; the automatic gain control (AGC) target, 2 × 10^5^; isolation window, 4.0 *m*/*z*; (N) CE/stepped (N) CE: 10, 30, 55 V. Xcalibur software version 2.07 (Thermo Fisher Scientific, USA) was used for the data acquisition and analysis.

### 3.3. Animals and Drug Administration

Male Sprague-Dawley rats (180–220 g) were obtained from the National Institutes for Food and Drug Control (Beijing, China). Before the experiment, all the rats were bred in a controlled breeding environment. All the experimental procedures in this study were performed following the Guide for Institute of Materia Medica, Chinese Academy of Medical Science. The rats were fasted for 24 h with free access to water before administration. Ilaprazole suspension in 0.5% CMC-Na was administered to the rats via gavage at a dose of 20 mg/kg.

### 3.4. Pretreatment of the Sample

Blood samples were taken from the angular vein after oral administration and put into heparinized Eppendorf tubes. Plasma samples were obtained via centrifugation at 4 °C for 10 min at 3500 r/min. The treated samples were stored at −80 °C until analysis. The plasma samples were then thawed at room temperature. Ethyl acetate and the plasma samples were added into a clean Eppendorf tube at a ratio of 6:1, vortexed for 5 min, and centrifuged for 10 min at 8000 r/min. The upper organic phase was transferred into a clean Eppendorf tube and dried with N_2_. The residues were reconstituted with 200 μL of a 10 mM ammonium acetate-methanol (2:8, *v*/*v*, adjusted to pH 9–10 with ammonia solution) and filtered through a 0.22 µm microporous membrane before the LC-MS analysis.

### 3.5. In Silico Bioactivity Prediction

PASS software can predict biochemical mechanisms and more than 300 pharmacological effects on the basis of the structural formula of a substance. A World Wide Web (WWW) server was used for the on-line prediction of the biological activity [17]. The potential bioactivities of the H^+^/K^+^-ATPase inhibitor were predicted by the PASS software product and the data of the prediction model were from Shanghai Institute of Organic Chemistry (SIOC).

## 4. Conclusions

A total of twelve metabolites of ilaprazole were detected in rat plasma after oral administration. The metabolites can be divided into three structural types: reductive metabolites with sulfide (**M1**, **M3**, **M4**, **M5**, and **M10**), oxidative metabolites with sulfone (**M2**, **M9**, **M11**, and **M12**), and hydroxylated metabolites with sulfoxide (**M6**, **M7**, and **M8**). The structures of the six new in vivo metabolites (**M3**, **M7**, **M8**, **M9**, **M11**, and **M12**) were identified and characterized via LC-HRMS and LC-MS^n^. The fragmentation behaviors of ilaprazole and its metabolites were concluded as follows. The characteristic fragments of ilaprazole and its metabolites were created after the fragmentation of the bond between C and S. The typical fragment ion losing HS·(33 Da) would form in the metabolites with the structure of sulfide. The metabolites with the structure of sulfone would produce a characteristic fragmentation behavior with a neutral loss of SO_2_ (64 Da). The fragmentation behaviors of ilaprazole and its metabolites were described in this research, which could be valuable in rapidly detecting the metabolism of similar structures. The results of the in silico prediction suggested that all the new ilaprazole metabolites have shown the potential ability to inhibit H^+^/K^+^-ATPase activity. This provides stronger support for the efficacy of ilaprazole and gives an important basis for the design of new PPIs.

## Figures and Tables

**Table 1:** In silico bioactivity prediction of the new metabolites.

Name	Probability of H^+^/K^+^-ATPase Inhibitor	Name	Probability of H^+^/K^+^-ATPase Inhibitor
**Ilaprazole**	94.1%	M8-3 *	68.4%
**M3**	60.9%	M9-1 *	88.2%
M7-1 *	89.4%	M9-2 *	73.9%
M7-2 *	76.5%	**M11**	84.3%
M8-1 *	79.1%	M12-1 *	84.0%
M8-2 *	90.7%	M12-2 *	66.1%

* The possible structure is shown in Supplementary Material Table S2.

## Data Availability

The data presented in this study are available on request from the corresponding author.

## Supplementary Materials

The following are available online, Table S1: Accurate mass data of ilaprazole and twelve metabolites. Table S2: The possible structure of the metabolites. Figure S1A: The MS^n^ spectra of Ilaprazole from [M + H]^+^ at *m*/*z* 367; Figure S1B: The MS^n^ spectrum of *m*/*z* 168 ion from [M + H]^+^ at *m*/*z* 351 of M1; Figure S1C: The MS^n^ spectrum of *m*/*z* 200 ion from [M + H]^+^ at *m*/*z* 383 of M2; Figure S1D: The MS^n^ spectra of M3 from [M + H]^+^ at *m*/*z* 343; Figure S1E: The MS^n^ spectra of M4 from [M + H]^+^ at *m*/*z* 367; Figure S1F: The MS^n^ spectra of M5 from [M + H]^+^ at *m/z* 383; Figure S1G: The MS^n^ spectra of M6 from [M + H]^+^ at *m*/*z* 383; Figure S1H: The MS^n^ spectra of M7 from [M + H]^+^ at *m*/*z* 383; Figure S1I: The MS^n^ spectra of M8 from [M + H]^+^ at *m*/*z* 399; Figure S1J: The MS^n^ spectra of M9 from [M + H]^+^ at *m*/*z* 399; Figure S1K: The MS^n^ spectra of M10 from [M + H]^+^ at *m*/*z* 399; Figure S1L: The MS^n^ spectrum of *m*/*z* 381 ion from [M + H]^+^ at *m*/*z* 399 of M11; Figure S1M: The MS^n^ spectrum of *m*/*z* 216 ion from [M + H]^+^ at *m*/*z* 399 of M12. Figure S2A: The HRMS/MS spectrum and the possible fragmentation pathway of M4; Figure S2B: The HRMS/MS spectrum and the possible fragmentation pathway of M5; Figure S2C: The HRMS/MS spectrum and the possible fragmentation pathway of M6; Figure S2D: The HRMS/MS spectrum and the possible fragmentation pathway of M10.

## Sample Availability

Not available.
