# Supplementary material for: Identification of the New In Vivo Metabolites of Ilaprazole in Rat Plasma after Oral Administration by LC-MS: In Silico Prediction of the H+/K+-ATPase Inhibitor
Source: Molecules. 2021 Jan 16;26(2):459. doi: 10.3390/molecules26020459 (PMC7829900; doi:10.3390/molecules26020459)
Supplement: Supplementary file 1 [file molecules-26-00459-s001.pdf]

Supplementary Materials

Table S1 Accurate mass data of ilaprazole and twelve metabolites.

	Observed mass	Calculated mass	Proposed molecular formula	Error（ppm）	Proposed loss
ilaprazole	367.1220	367.1223	$C_{19}H_{19}N_4O_2S^+$	−0.82	
	349.1113	349.1118	$C_{19}H_{17}N_4OS^+$	−1.43	$-H_2O$
	214.0432	214.0433	$C_{11}H_8N_3S^+$	−0.47	$-C_8H_{11}NO_2$
	184.0865	184.0869	$C_{11}H_{10}N_3^+$	−2.17	$-C_8H_9NO_2S$
	184.0426	184.0427	$C_8H_{10}NO_2S^+$	−0.54	$-C_{11}H_9N_3$
	166.0320	166.0321	$C_8H_8NOS^+$	−0.60	$-C_{11}H_9N_3-H_2O$
	154.0862	154.0863	$C_8H_{12}NO_2^+$	−0.65	$-C_{11}H_7N_3S$
	137.0835	137.0835	$C_8H_{11}NO^{\cdot+}$	0.00	$-C_{11}H_8N_3OS\cdot$
	136.0757	136.0757	$C_8H_{10}NO^+$	0.00	$-C_{11}H_7N_3S-H_2O$
	122.0602	122.0600	$C_7H_8NO^+$	1.64	$-C_{12}H_{11}N_3OS$
**M1**	351.1270	351.1274	$C_{19}H_{19}N_4OS^+$	−1.14	
	318.1471	318.1475	$C_{19}H_{18}N_4O^{\cdot+}$	−1.26	$-HS\cdot$
	184.0869	184.0869	$C_{11}H_{10}N_3^+$	0.00	$-C_8H_9NOS$
	168.0476	168.0478	$C_8H_{10}NOS^+$	−1.19	$-C_{11}H_9N_3$
	137.0836	137.0835	$C_8H_{11}NO^{\cdot+}$	0.73	$-C_{11}H_8N_3S\cdot$
	136.0757	136.0757	$C_8H_{10}NO^+$	0.00	$-C_{11}H_9N_3-S$
	122.0602	122.0600	$C_7H_8NO^+$	1.64	$-C_{12}H_{11}N_3S$
**M2**	383.1168	383.1172	$C_{19}H_{19}N_4O_3S^+$	−1.04	
	319.1550	319.1553	$C_{19}H_{19}N_4O^+$	−0.94	$-SO_2$
	230.0381	230.0383	$C_{11}H_8N_3OS^+$	−0.87	$-C_8H_{11}NO_2$
	200.0376	200.0376	$C_8H_{10}NO_3S^+$	0.00	$-C_{11}H_9N_3$
	184.0869	184.0869	$C_{11}H_{10}N_3^+$	0.00	$-C_8H_9NO_3S$
	152.0706	152.0706	$C_8H_{10}NO_2^+$	0.00	$-C_{11}H_9N_3-SO$
	137.0837	137.0835	$C_8H_{11}NO^{\cdot+}$	1.46	$-C_{11}H_8N_3O_2S\cdot$
	136.0757	136.0757	$C_8H_{10}NO^+$	0.00	$-C_{11}H_9N_3-SO_2$
	122.0602	122.0600	$C_7H_8NO^+$	1.64	$-C_{12}H_{11}N_3O_2S$
**M3**	343.1215	343.1223	$C_{17}H_{19}N_4O_2S^+$	−2.33	
	310.1415	310.1424	$C_{17}H_{18}N_4O_2^{\cdot+}$	−2.90	$-HS\cdot$
	176.0815	176.0818	$C_9H_{10}N_3O^+$	−1.70	$-C_8H_9NOS$
	168.0474	168.0478	$C_8H_{10}NOS^+$	−2.38	$-C_9H_9N_3O$
	137.0834	137.0835	$C_8H_{11}NO^{\cdot+}$	−0.73	$-C_9H_8N_3OS\cdot$
	136.0755	136.0757	$C_8H_{10}NO^+$	−1.47	$-C_9H_9N_3O-S$
	134.0708	134.0713	$C_7H_8N_3^+$	−3.73	$-C_8H_9NOS-C_2H_2O$
	122.0600	122.0600	$C_7H_8NO^+$	0.00	$-C_{10}H_{11}N_3OS$
**M4**	367.1219	367.1223	$C_{19}H_{19}N_4O_2S^+$	−1.09	
	334.1417	334.1424	$C_{19}H_{18}N_4O_2^{\cdot+}$	−2.09	$-HS\cdot$
	200.0817	200.0818	$C_{11}H_{10}N_3O^+$	−0.50	$-C_8H_9NOS$
	172.0868	172.0869	$C_{10}H_{10}N_3^+$	−0.58	$-C_8H_9NOS-CO$
	168.0476	168.0478	$C_8H_{10}NOS^+$	−1.19	$-C_{11}H_9N_3O$
	137.0836	137.0835	$C_8H_{11}NO^{\cdot+}$	0.73	$-C_{11}H_8N_3OS\cdot$

	136.0756	136.0757	$C_8H_{10}NO^+$	−0.73	$-C_{11}H_9N_3O$-S
	122.0602	122.0600	$C_7H_8NO^+$	1.64	$-C_{12}H_{11}N_3OS$
**M5**	383.1161	383.1172	$C_{19}H_{19}N_4O_3S^+$	−2.87	
	365.1055	365.1067	$C_{19}H_{17}N_4O_2S^+$	−3.29	$-H_2O$
	350.1363	350.1373	$C_{19}H_{18}N_4O_3^{\cdot+}$	−2.86	-HS·
	216.0763	216.0768	$C_{11}H_{10}N_3O_2^+$	−2.31	$-C_8H_9NOS$/ -HS·$-C_8H_8NO$·
	188.0816	188.0818	$C_{10}H_{10}N_3O^+$	−1.06	$-C_8H_9NOS$-CO
	168.0474	168.0478	$C_8H_{10}NOS^+$	−2.38	$-C_{11}H_9N_3O_2$
	137.0834	137.0835	$C_8H_{11}NO^{\cdot+}$	−0.73	$-C_{11}H_8N_3O_2S$·
	136.0755	136.0757	$C_8H_{10}NO^+$	−1.47	$-C_{11}H_9N_3O_2$-S
	122.0600	122.0600	$C_7H_8NO^+$	0.00	$-C_{12}H_{11}N_3O_2S$
**M6**	383.1169	383.1172	$C_{19}H_{19}N_4O_3S^+$	−0.78	
	365.1061	365.1067	$C_{19}H_{17}N_4O_2S^+$	−1.64	$-H_2O$
	230.0381	230.0383	$C_{11}H_8N_3OS^+$	−0.87	$-C_8H_{11}NO_2$
	200.0817	200.0818	$C_{11}H_{10}N_3O^+$	−0.50	$-C_8H_9NO_2S$
	184.0426	184.0427	$C_8H_{10}NO_2S^+$	−0.54	$-C_{11}H_9N_3O$
	172.0866	172.0869	$C_{10}H_{10}N_3^+$	−1.74	$-C_8H_9NO_2S$-CO
	166.0320	166.0321	$C_8H_8NOS^+$	−0.60	$-C_{11}H_9N_3O-H_2O$
	154.0862	154.0863	$C_8H_{12}NO_2^+$	−0.65	$-C_{11}H_7N_3OS$
	137.0836	137.0835	$C_8H_{11}NO^{\cdot+}$	0.73	$-C_{11}H_8N_3O_2S$·
	136.0757	136.0757	$C_8H_{10}NO^+$	0.00	$-C_{11}H_7N_3OS-H_2O$
	122.0602	122.0600	$C_7H_8NO^+$	1.64	$-C_{12}H_{11}N_3O_2S$
**M7**	383.1172	383.1172	$C_{19}H_{19}N_4O_3S^+$	0.00	
	214.0434	214.0433	$C_{11}H_8N_3S^+$	0.47	$-C_8H_{11}NO_3$
	200.0376	200.0376	$C_8H_{10}NO_3S^+$	0.00	$-C_{11}H_9N_3$
	184.0868	184.0869	$C_{11}H_{10}N_3^+$	−0.55	$-C_8H_9NO_3S$
	182.0269	182.0270	$C_8H_8NO_2S^+$	−0.54	$-C_{11}H_9N_3-H_2O$
	170.0811	170.0812	$C_8H_{12}NO_3^+$	−0.55	$-C_{11}H_7N_3S$
	154.0321	154.0321	$C_7H_8NOS^+$	−0.59	$-C_{11}H_9N_3-H_2O$-CO
	153.0784	153.0784	$C_8H_{11}NO_2^{\cdot+}$	0.00	$-C_{11}H_8N_3OS$·
	152.0706	152.0706	$C_8H_{10}NO_2^+$	0.00	$-C_{11}H_7N_3S-H_2O$
	138.0549	138.0550	$C_7H_8NO_2^+$	−0.72	$-C_{12}H_{11}N_3OS$
	124.0759	124.0757	$C_7H_{10}NO^+$	1.61	$-C_{11}H_7N_3S-H_2O$-CO
**M8**	399.1114	399.1122	$C_{19}H_{19}N_4O_4S^+$	−2.00	
	381.1010	381.1016	$C_{19}H_{17}N_4O_3S^+$	−1.57	$-H_2O$
	246.0323	246.0332	$C_{11}H_8N_3O_2S^+$	−3.66	$-C_8H_{11}NO_2$
	216.0761	216.0768	$C_{11}H_{10}N_3O_2^+$	−3.24	$-C_8H_9NO_2S$
	200.0816	200.0818	$C_{11}H_{10}N_3O^+$	−1.00	$-C_8H_9NO_3S$
	198.0657	198.0662	$C_{11}H_8N_3O^+$	−2.52	$-C_8H_9NO_2S-H_2O$
	188.0816	188.0818	$C_{10}H_{10}N_3O^+$	−1.06	$-C_8H_9NO_2S$-CO
	184.0428	184.0427	$C_8H_{10}NO_2S^+$	0.54	$-C_{11}H_9N_3O_2$
	172.0864	172.0869	$C_{10}H_{10}N_3^+$	−2.91	$-C_8H_9NO_3S$-CO
	166.0319	166.0321	$C_8H_8NOS^+$	−1.20	$-C_{11}H_9N_3O_2-H_2O$
	154.0859	154.0863	$C_8H_{12}NO_2^+$	−2.60	$-C_{11}H_7N_3O_2S$
	137.0837	137.0835	$C_8H_{11}NO^{\cdot+}$	1.46	$-C_{11}H_8N_3O_3S$·

	136.0754	136.0757	$C_8H_{10}NO^+$	−2.20	$-C_{11}H_7N_3O_2S-H_2O$
	122.0602	122.0600	$C_7H_8NO^+$	1.64	$-C_{12}H_{11}N_3O_3S$
**M9**	399.1118	399.1122	$C_{19}H_{19}N_4O_4S^+$	−1.00	
	335.1500	335.1503	$C_{19}H_{19}N_4O_2^+$	−0.90	$-SO_2$
	307.1549	307.1553	$C_{18}H_{19}N_4O^+$	−1.30	$-SO_2-CO$
	246.0329	246.0332	$C_{11}H_8N_3O_2S^+$	−1.22	$-C_8H_{11}NO_2$
	200.0817	200.0818	$C_{11}H_{10}N_3O^+$	1.50	$-C_8H_9NO_3S$
	200.0379	200.0376	$C_8H_{10}NO_3S^+$	−0.50	$-C_{11}H_9N_3O$
	172.0868	172.0869	$C_{10}H_{10}N_3^+$	−0.58	$-C_8H_9NO_3S-CO$
	152.0706	152.0706	$C_8H_{10}NO_2^+$	0.00	$-C_{11}H_9N_3O-SO$
	137.0836	137.0835	$C_8H_{11}NO^{\cdot+}$	0.73	$-C_{11}H_8N_3O_3S\cdot$
	136.0757	136.0757	$C_8H_{10}NO^+$	0.00	$-C_{11}H_9N_3O-SO_2$
	122.0602	122.0600	$C_7H_8NO^+$	1.64	$-C_{12}H_{11}N_3O_3S$
**M10**	399.1115	399.1122	$C_{19}H_{19}N_4O_4S^+$	−1.75	
	366.1312	366.1323	$C_{19}H_{18}N_4O_4^{\cdot+}$	−3.00	$-HS\cdot$
	232.0712	232.0717	$C_{11}H_{10}N_3O_3^+$	−2.15	$-C_8H_9NOS$
	214.0608	214.0611	$C_{11}H_8N_3O_2^+$	−1.40	$-C_8H_9NOS-H_2O$
	186.0658	186.0662	$C_{10}H_8N_3O^+$	−2.15	$-C_8H_9NOS- H_2O-CO$
	168.0476	168.0478	$C_8H_{10}NOS^+$	−1.19	$-C_{11}H_9N_3O_3$
	137.0836	137.0835	$C_8H_{11}NO^{\cdot+}$	0.73	$-C_{11}H_8N_3O_3S\cdot$
	136.0757	136.0757	$C_8H_{10}NO^+$	0.00	$-C_{11}H_9N_3O_3-S$
	134.0707	134.0713	$C_7H_8N_3^+$	−4.48	$-C_8H_9NOS-C_4H_2O_3$
	122.0602	122.0600	$C_7H_8NO^+$	1.64	$-C_{12}H_{11}N_3O_3S$
**M11**	399.1116	399.1122	$C_{19}H_{19}N_4O_4S^+$	−1.50	
	381.1011	381.1016	$C_{19}H_{17}N_4O_3S^+$	−1.31	$-H_2O$
	317.1393	317.1397	$C_{19}H_{17}N_4O^+$	−1.26	$-H_2O-SO_2$
	301.1081	301.1084	$C_{18}H_{13}N_4O^+$	−1.00	$-CH_6O_3S$
	216.0326	216.0325	$C_8H_{10}NO_4S^+$	0.46	$-C_{11}H_9N_3$
	184.0869	184.0869	$C_{11}H_{10}N_3^+$	0.00	$-C_8H_9NO_4S$
	138.0549	138.0550	$C_7H_8NO_2^+$	−0.72	$-C_{12}H_{11}N_3O_2S$
**M12**	399.1118	399.1122	$C_{19}H_{19}N_4O_4S^+$	−1.00	
	216.0324	216.0325	$C_8H_{10}NO_4S^+$	−0.46	$-C_{11}H_9N_3$
	184.0868	184.0869	$C_{11}H_{10}N_3^+$	−0.54	$-C_8H_9NO_4S$
	152.0706	152.0706	$C_8H_{10}NO_2^+$	0.00	$-C_{11}H_9N_3-SO_2$
	140.0706	140.0705	$C_7H_{10}NO_2^+$	0.71	$-C_{12}H_9N_3O_2S$

Table S2 The possible structure of the metabolites

Name	Structure	Name	Structure
**M7-1**	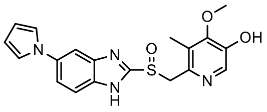	**M9-1**	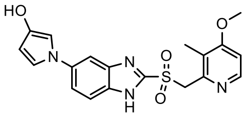
**M7-2**	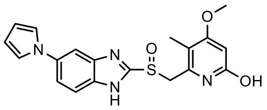	**M9-2**	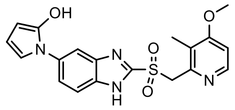
**M8-1**	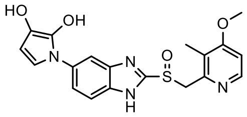	**M12-1**	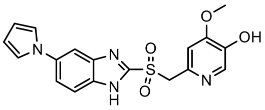
**M8-2**	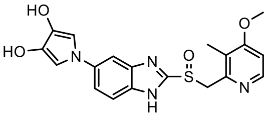	**M12-2**	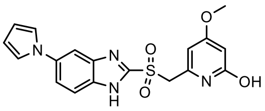
**M8-3**	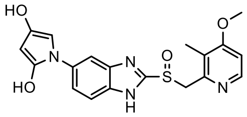		

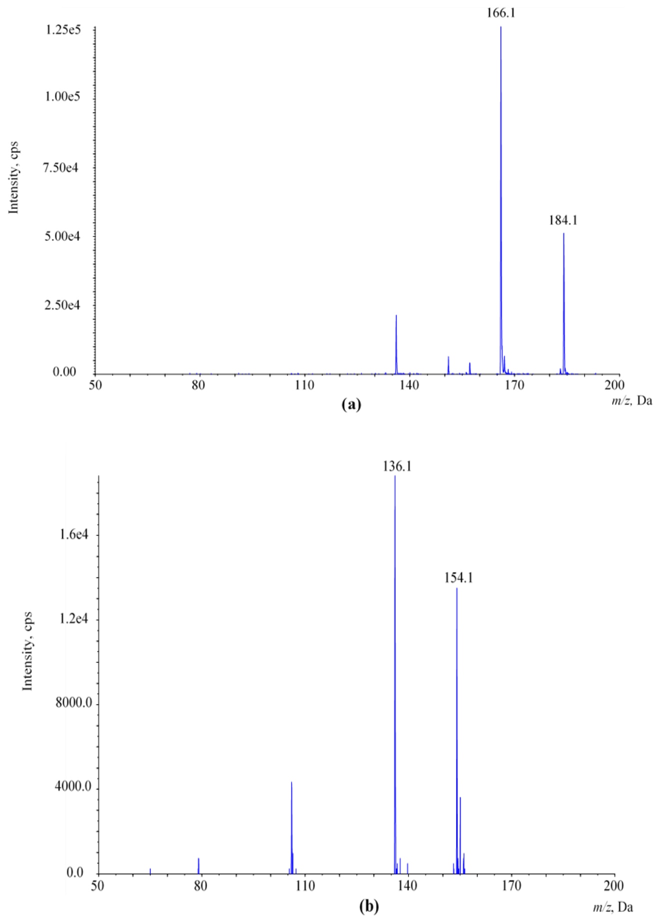

Figure S1A. The MS^n spectra of Ilaprazole from $[M + H]^+$ at *m/z* 367.

(a) The MS^n spectrum of *m/z* 184 ion from $[M + H]^+$ at *m/z* 367

(b) The MS^n spectrum of *m/z* 154 ion from $[M + H]^+$ at *m/z* 367

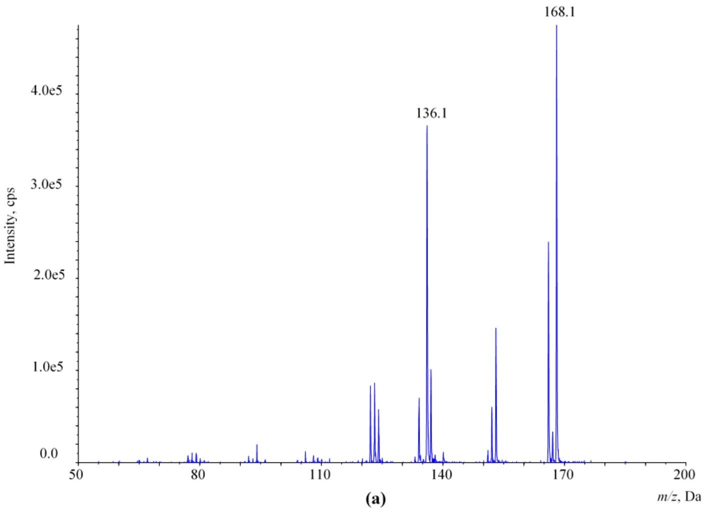

Figure S1B.. The MS^n spectrum of *m/z* 168 ion from $[M + H]^+$ at *m/z* 351 of M1.

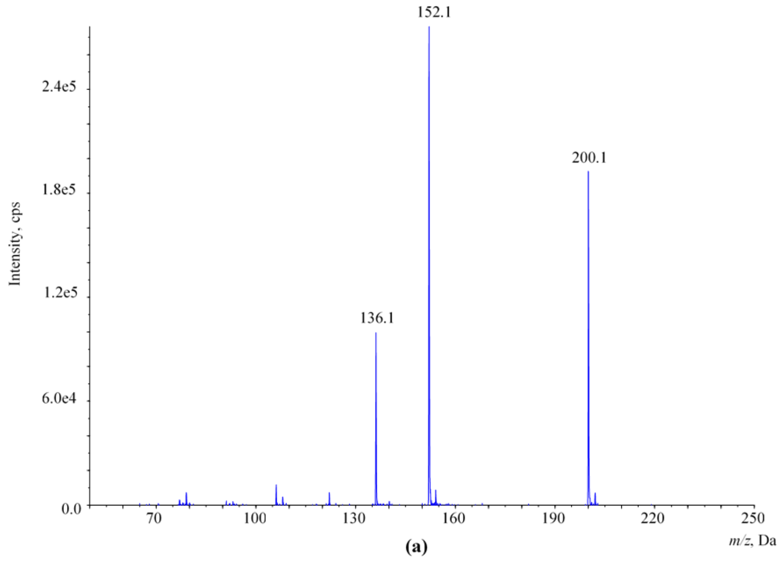

Figure S1C. The MS^n spectrum of *m/z* 200 ion from [M + H]$^+$ at *m/z* 383 of M2.

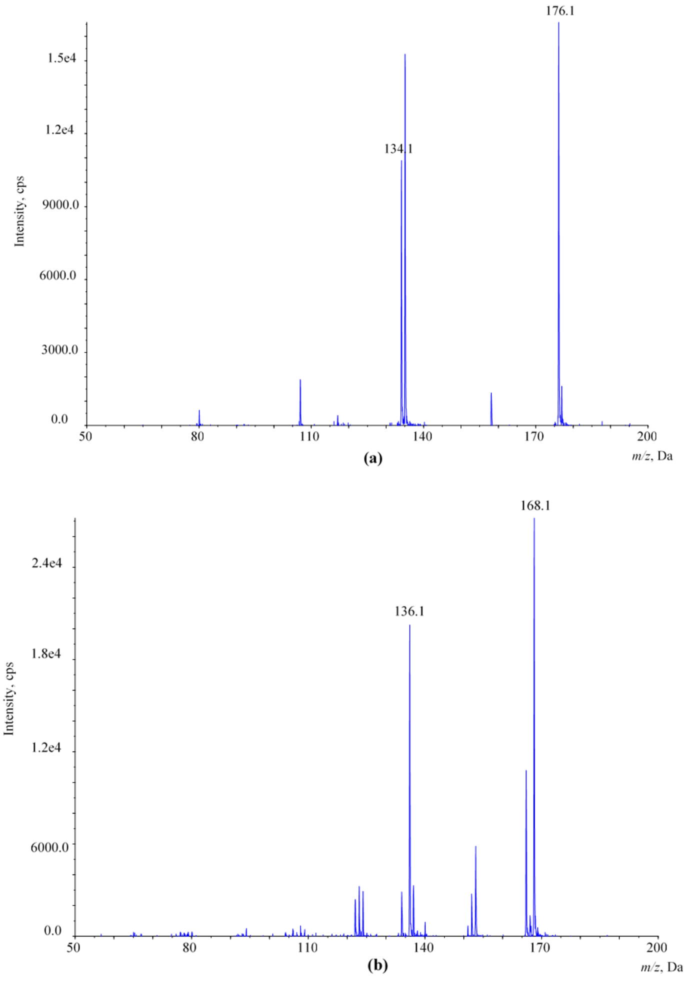

Figure S1D. The MS^n spectra of M3 from [M + H]$^+$ at *m/z* 343.

(a) The MS^n spectrum of *m/z* 176 ion from [M + H]$^+$ at *m/z* 343

(b) The MS^n spectrum of *m/z* 168 ion from [M + H]$^+$ at *m/z* 343

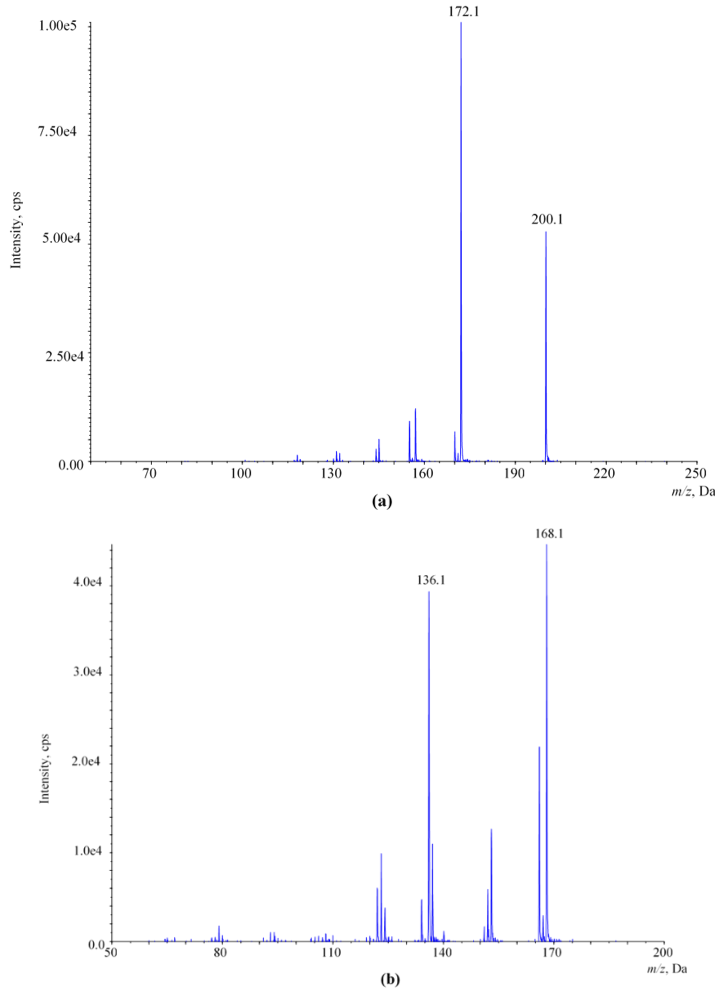

Figure S1E. The MS^n spectra of M4 from $[M + H]^+$ at *m/z* 367.

(a) The MS^n spectrum of *m/z* 200 ion from $[M + H]^+$ at *m/z* 367

(b) The MS^n spectrum of *m/z* 168 ion from $[M + H]^+$ at *m/z* 367

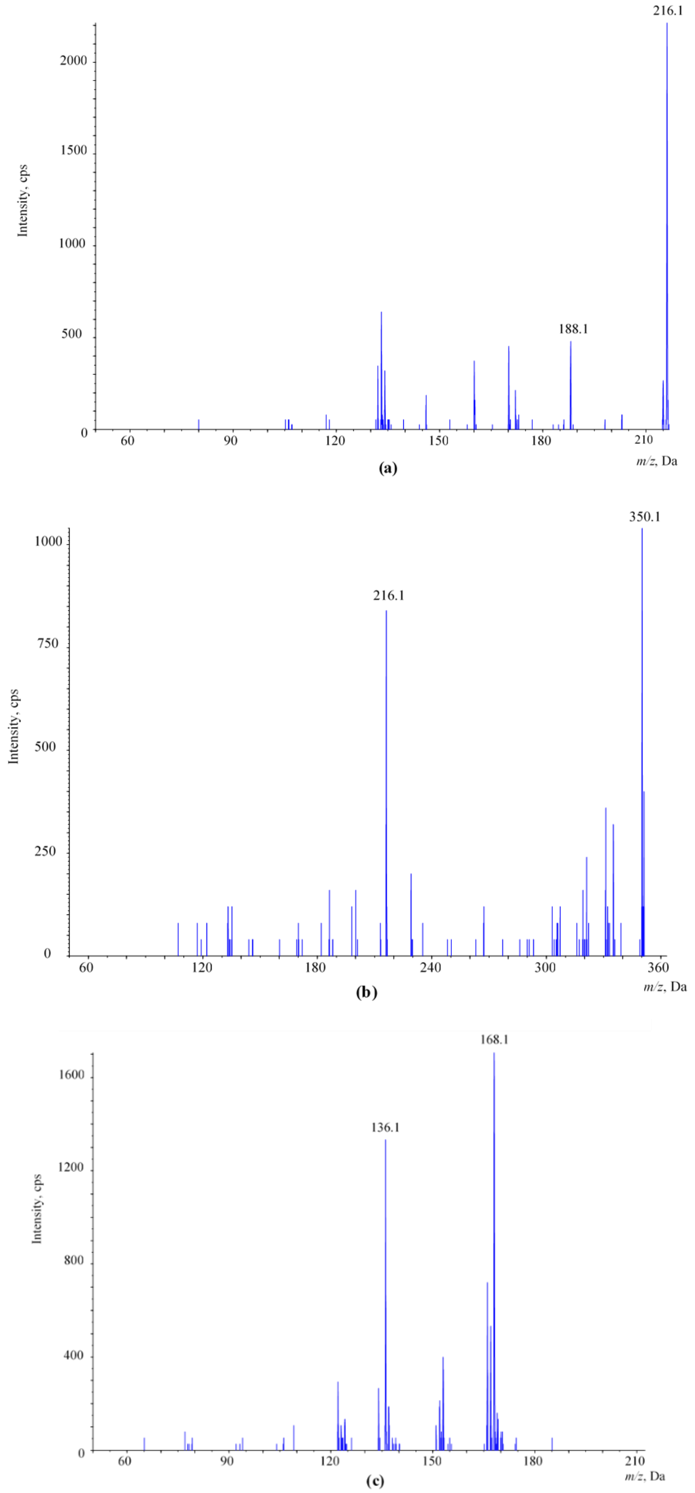

Figure S1F. The MS^n spectra of M5 from $[M + H]^+$ at *m/z* 383.

(a) The MS^n spectrum of *m/z* 216 ion from $[M + H]^+$ at *m/z* 383

(b) The MS^n spectrum of *m/z* 350 ion from $[M + H]^+$ at *m/z* 383

(c) The MS^n spectrum of *m/z* 168 ion from $[M + H]^+$ at *m/z* 383

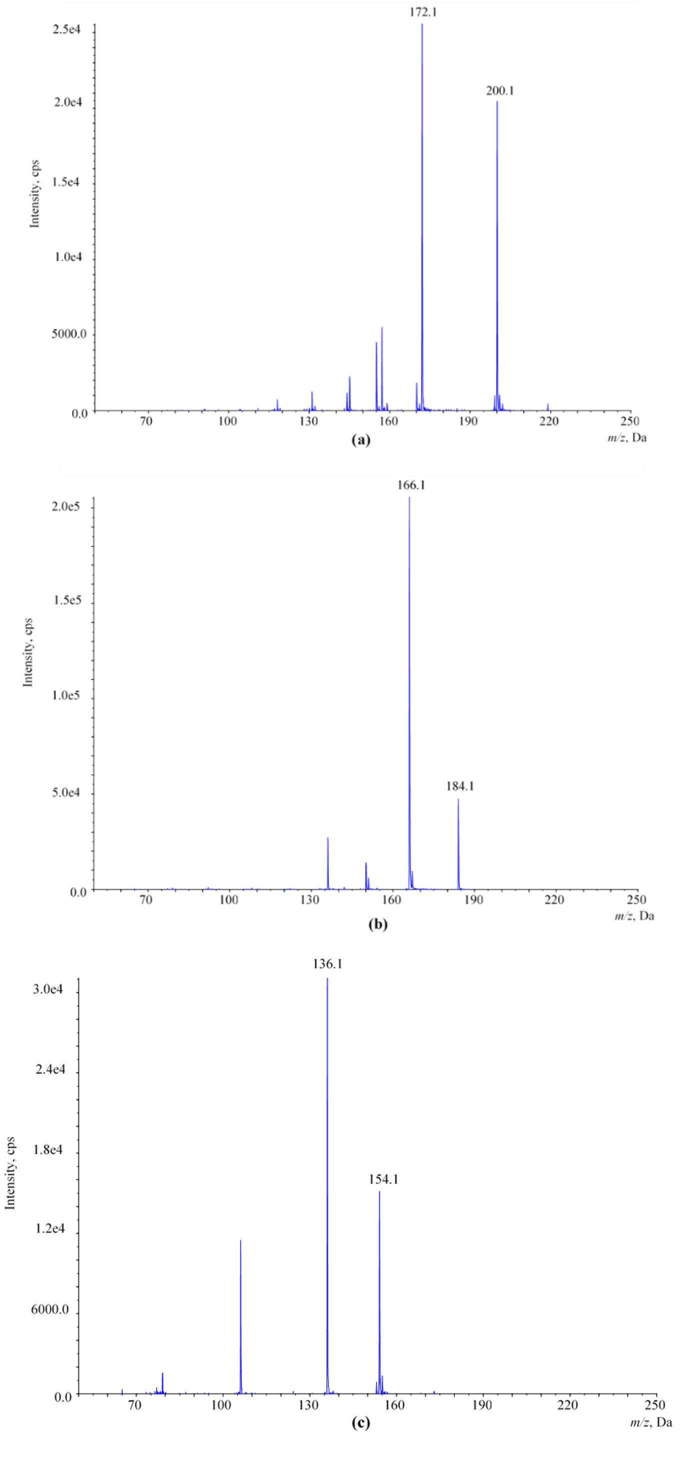

Figure S1G. The MS^n spectra of M6 from [M+H]$^+$ at *m/z* 383.

(a) The MS^n spectrum of *m/z* 200 ion from [M + H]$^+$ at *m/z* 383

(b) The MS^n spectrum of *m/z* 184 ion from [M + H]$^+$ at *m/z* 383

(c) The MS^n spectrum of *m/z* 154 ion from [M + H]$^+$ at *m/z* 383

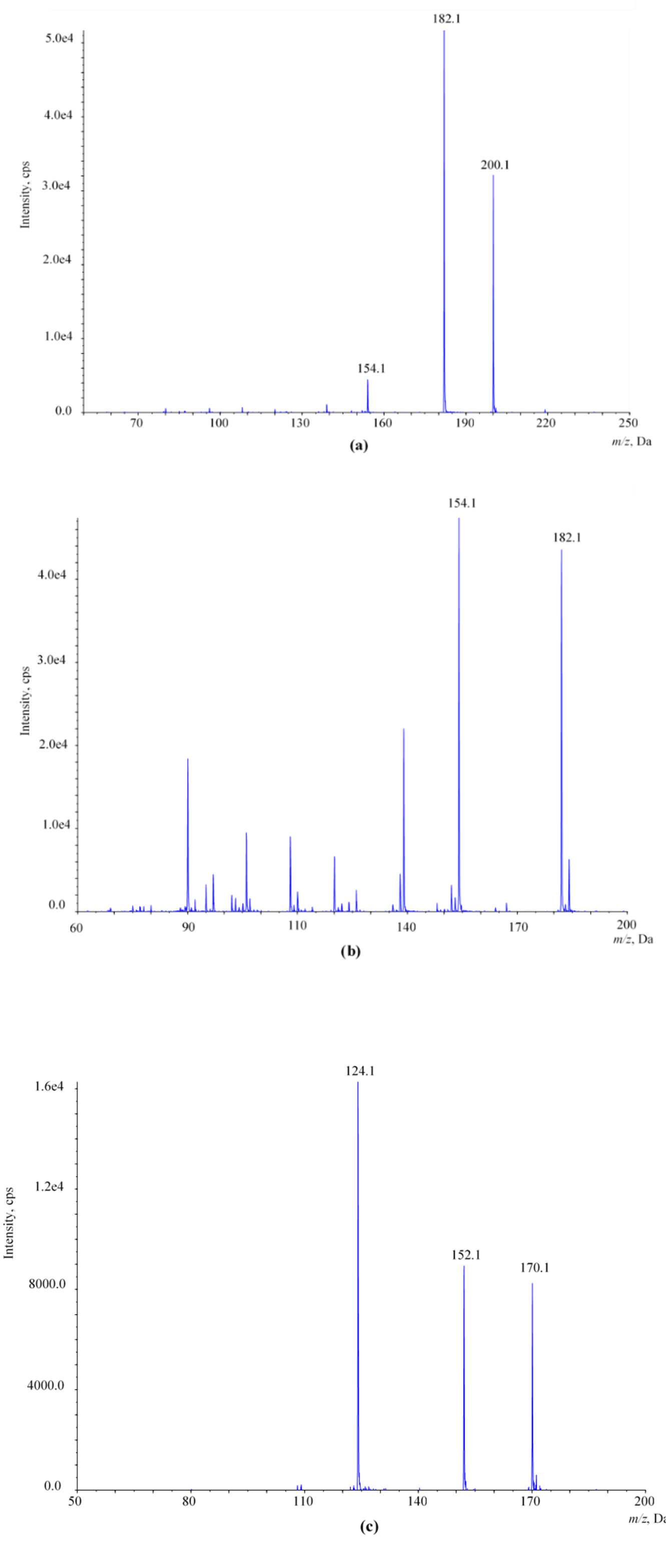

Figure S1H. The MS^n spectra of M7 from $[M + H]^+$ at *m/z* 383.

(a) The MS^n spectrum of *m/z* 200 ion from $[M + H]^+$ at *m/z* 383

(b) The MS^n spectrum of *m/z* 182 ion from $[M + H]^+$ at *m/z* 383

(c) The MS^n spectrum of *m/z* 170 ion from $[M + H]^+$ at *m/z* 383

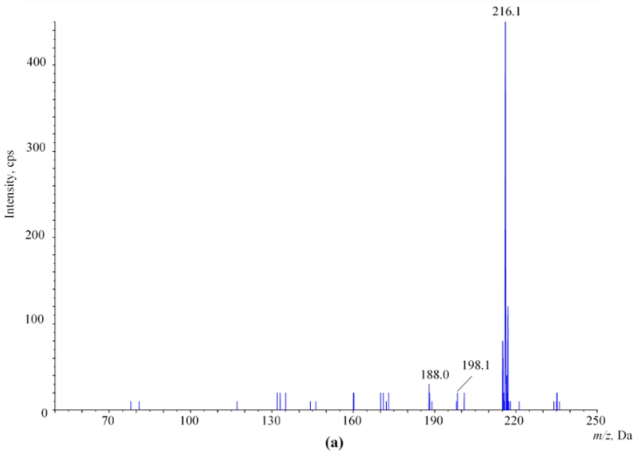
216.1
188.0
198.1
Intensity, cps
0
100
200
300
400
70
100
130
160
190
220
250
m/z, Da

(a)

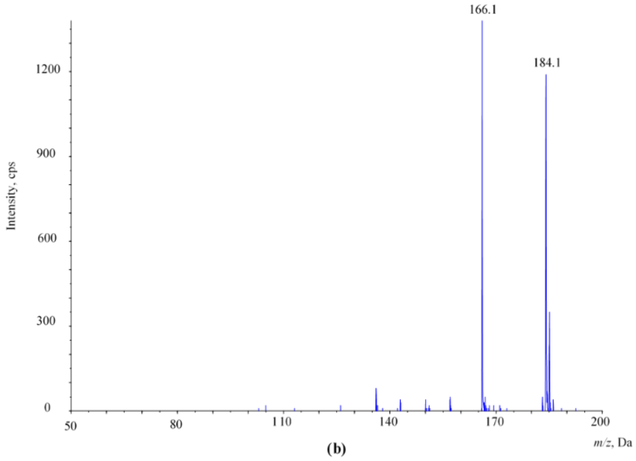
166.1
184.1
Intensity, cps
0
300
600
900
1200
50
80
110
140
170
200
m/z, Da

(b)

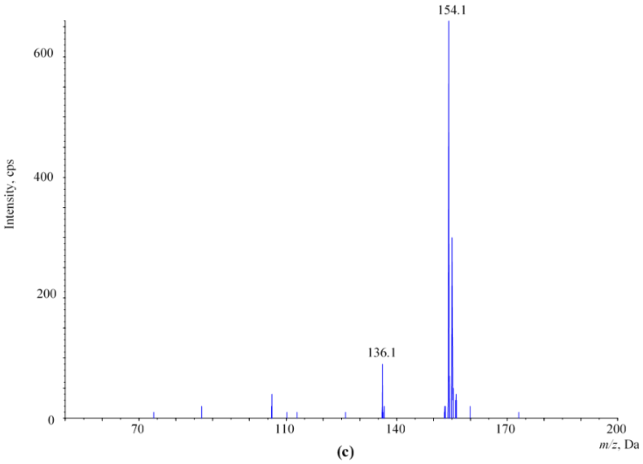
154.1
136.1
Intensity, cps
0
200
400
600
70
110
140
170
200
m/z, Da

(c)

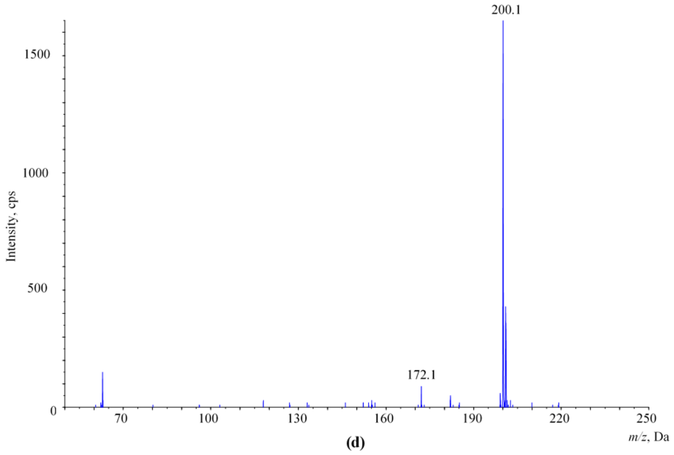
200.1
172.1
Intensity, cps
0
500
1000
1500
70
100
130
160
190
220
250
m/z, Da

(d)

Figure S1I. The MS^n spectra of M8 from $[M + H]^+$ at *m/z* 399.

(a) The MS^n spectrum of *m/z* 216 ion from $[M + H]^+$ at *m/z* 399

(b) The MS^n spectrum of *m/z* 184 ion from $[M + H]^+$ at *m/z* 399

(c) The MS^n spectrum of *m/z* 154 ion from $[M + H]^+$ at *m/z* 399

(d) The MS^n spectrum of *m/z* 200 ion from $[M + H]^+$ at *m/z* 399

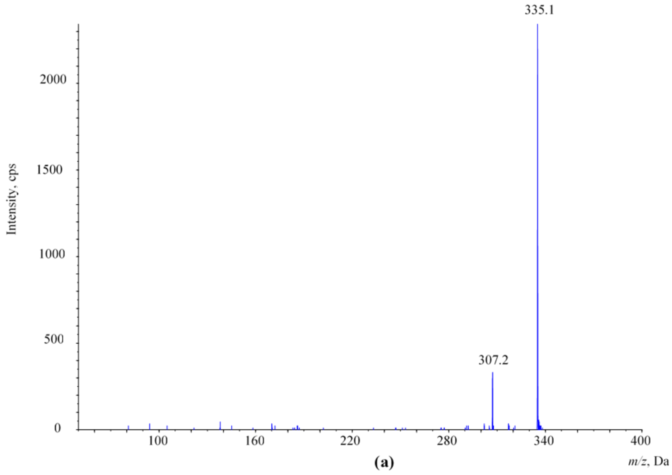

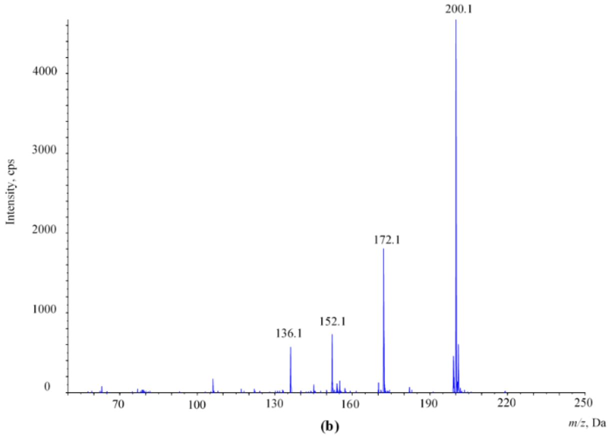

Figure S1J. The MS^n spectra of M9 from $[M + H]^+$ at *m/z* 399.

(a) The MS^n spectrum of *m/z* 335 ion from $[M + H]^+$ at *m/z* 399

(b) The MS^n spectrum of *m/z* 200 ion from $[M + H]^+$ at *m/z* 399

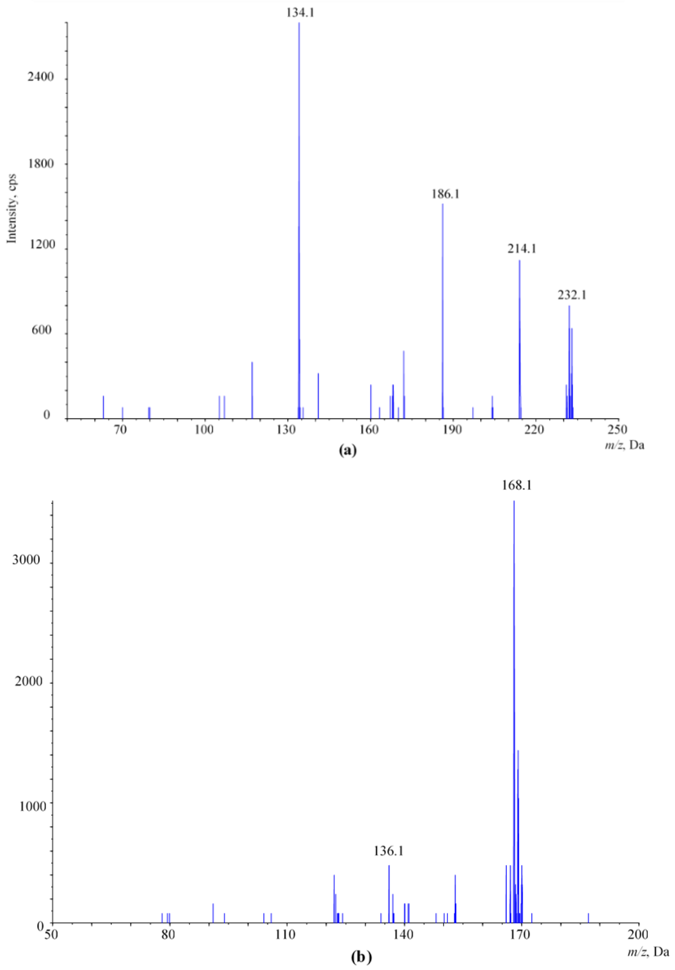

Figure S1K. The MS^n spectra of M10 from [M + H]$^+$ at *m/z* 399.

(a) The MS^n spectrum of *m/z* 232 ion from [M + H]$^+$ at *m/z* 399

(b) The MS^n spectrum of *m/z* 168 ion from [M + H]$^+$ at *m/z* 399

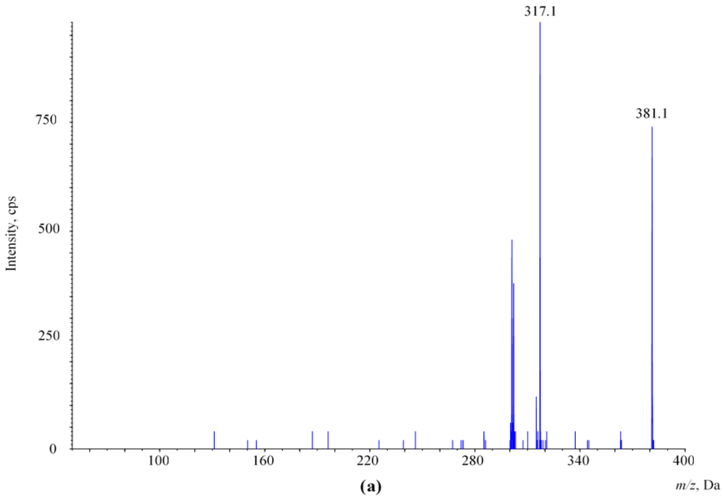

Figure S1L. The MS^n spectrum of *m/z* 381 ion from [M + H]$^+$ at *m/z* 399 of M11.

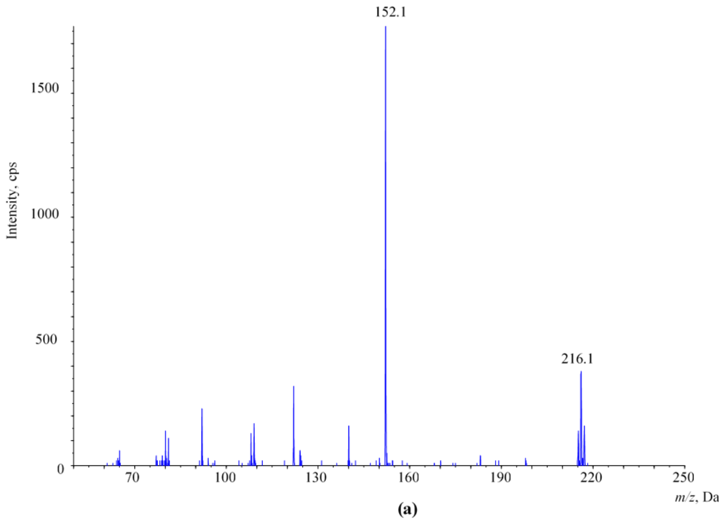

Figure S1M. The MS^n spectrum of *m/z* 216 ion from $[M + H]^+$ at *m/z* 399 of M12.

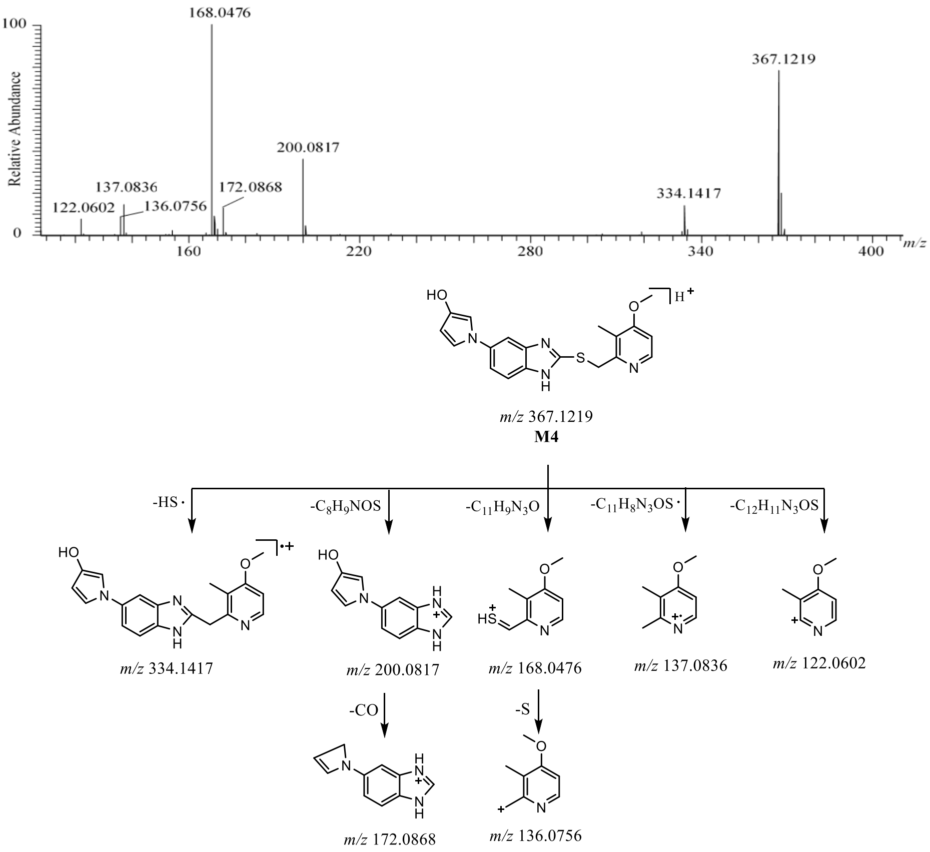

Figure S2A. The HRMS/MS spectrum and the possible fragmentation pathway of M4

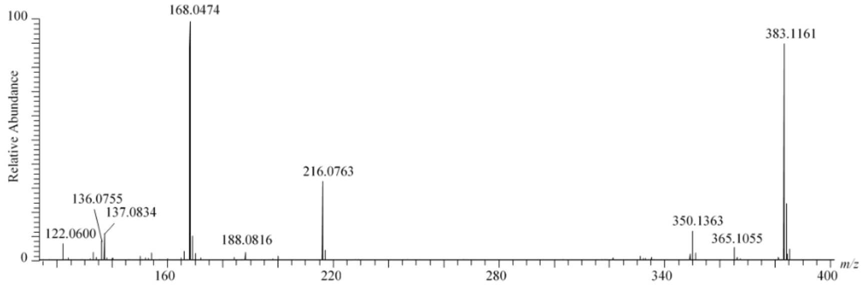

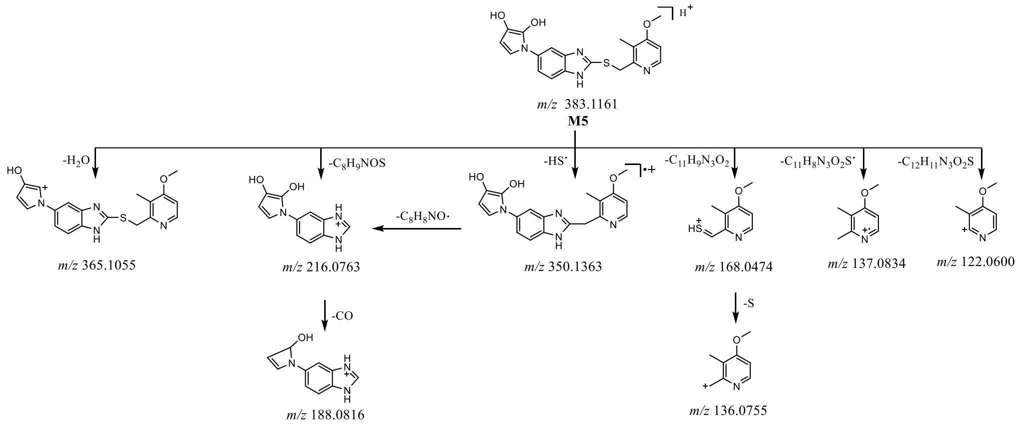

Figure S2B. The HRMS/MS spectrum and the possible fragmentation pathway of M5

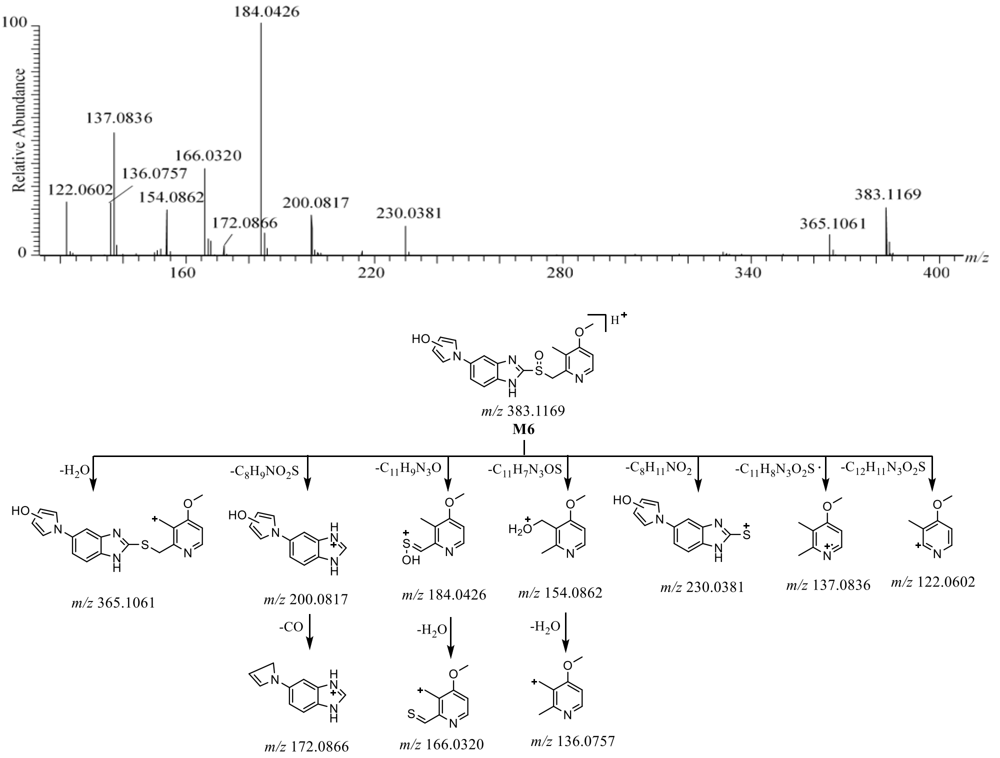

Figure S2C. The HRMS/MS spectrum and the possible fragmentation pathway of M6

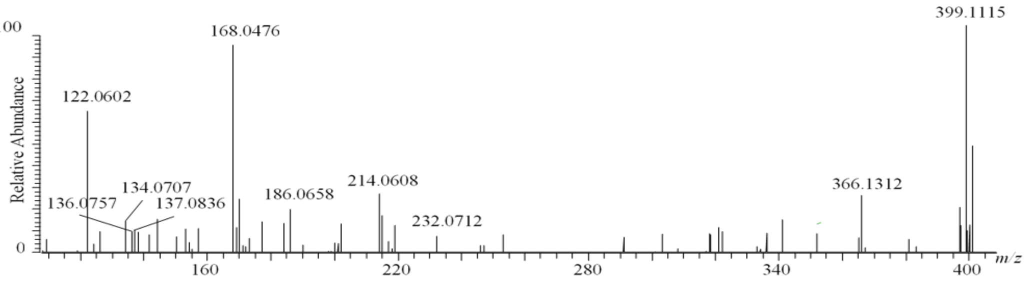

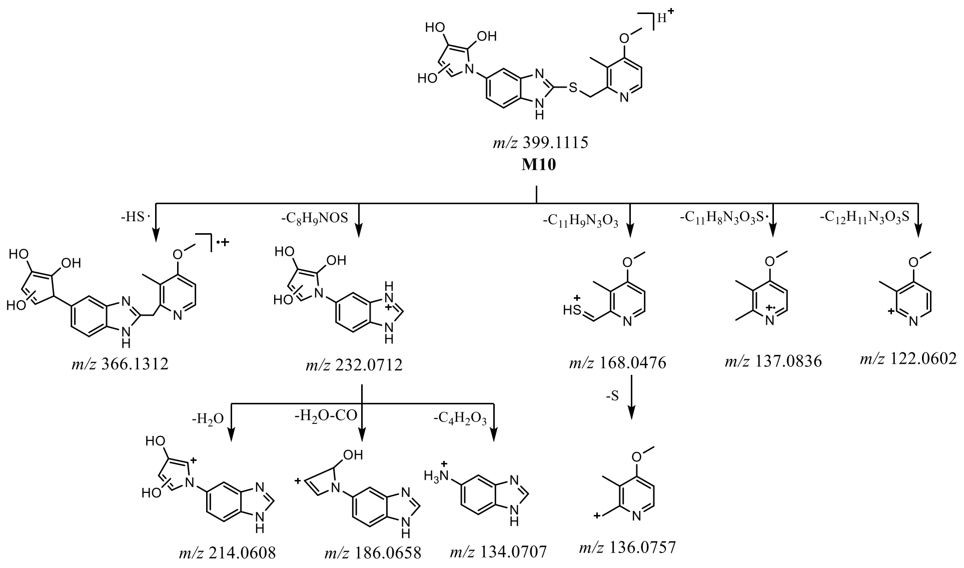

Figure S2D. The HRMS/MS spectrum and the possible fragmentation pathway of M10
